# Characterization of FUS Mutations in Amyotrophic Lateral Sclerosis Using RNA-Seq

**DOI:** 10.1371/journal.pone.0060788

**Published:** 2013-04-08

**Authors:** Marka van Blitterswijk, Eric T. Wang, Brad A. Friedman, Pamela J. Keagle, Patrick Lowe, Ashley Lyn Leclerc, Leonard H. van den Berg, David E. Housman, Jan H. Veldink, John E. Landers

**Affiliations:** 1 Department of Neurology, Rudolf Magnus Institute of Neuroscience, University Medical Center Utrecht, Utrecht, The Netherlands; 2 Department of Neurology, University of Massachusetts Medical Center, Worcester, Massachusetts, United States of America; 3 Department of Biology, Massachusetts Institute of Technology, Cambridge, Massachusetts, United States of America; 4 Harvard-MIT Division of Health Sciences and Technology, Cambridge, Massachusetts, United States of America; 5 Department of Molecular and Cell Biology, Harvard University, Cambridge, Massachusetts, United States of America; 6 David H. Koch Institute for Integrative Cancer Research, Massachusetts Institute of Technology, Cambridge, Massachusetts, United States of America; National Institute of Health, United States of America

## Abstract

Amyotrophic lateral sclerosis (ALS) is a neurodegenerative disease resulting in severe muscle weakness and eventual death by respiratory failure. Although little is known about its pathogenesis, mutations in fused in sarcoma/translated in liposarcoma (*FUS*) are causative for familial ALS. FUS is a multifunctional protein that is involved in many aspects of RNA processing. To elucidate the role of FUS in ALS, we overexpressed wild-type and two mutant forms of *FUS* in HEK-293T cells, as well as knocked-down *FUS* expression. This was followed by RNA-Seq to identify genes which displayed differential expression or altered splicing patterns. Pathway analysis revealed that overexpression of wild-type *FUS* regulates ribosomal genes, whereas knock-down of *FUS* additionally affects expression of spliceosome related genes. Furthermore, cells expressing mutant *FUS* displayed global transcription patterns more similar to cells overexpressing wild-type *FUS* than to the knock-down condition. This observation suggests that FUS mutants do not contribute to the pathogenesis of ALS through a loss-of-function. Finally, our results demonstrate that the R521G and R522G mutations display differences in their influence on transcription and splicing. Taken together, these results provide additional insights into the function of FUS and how mutations contribute to the development of ALS.

## Introduction

Amyotrophic lateral sclerosis (ALS) is a fatal neurodegenerative disease affecting upper and lower motor neurons causing progressive muscle weakness. Patients typically die within three to five years after onset of symptoms due to respiratory failure [1]. Although most cases are sporadic, approximately 10% of ALS cases are familial (FALS). Mutations in several genes are causative for FALS, including fused in sarcoma/translated in liposarcoma (*FUS*), superoxide dismutase-1 (*SOD1*), TAR DNA-binding protein (*TARDBP*), angiogenin (*ANG*), vesicle-associated membrane protein B (*VAPB*), optineurin (*OPTN*), and valosin-containing protein (*VCP*) [2], [3]. Recently, an expanded hexanucleotide repeat (GGGGCC) within chromosome 9 open reading frame 72 (*C9ORF72*) has also been identified in a large percentage (23.5–46%) of patients with ALS and frontotemporal dementia (FTD), and additionally, mutations in profilin 1 (*PFN1*) have been reported in patients with ALS [4]–[6].

Mutations in *FUS* are detected in ∼4% of FALS patients and infrequently in sporadic ALS (SALS) cases [7], [8]. FUS is also known as heterogeneous nuclear ribonucleoprotein (hnRNP) P2 and is involved in numerous aspects of RNA processing [8], [9]. The FUS protein is 526 amino acids long and contains an N-terminal serine, tyrosine, glycine and glutamine (SYGQ)-rich region, an RNA-recognition motif (RRM), a C2/C2 zinc finger motif, multiple arginine, glycine, and glycine (RGG)-repeat regions and a nuclear localization signal (NLS) at the extreme C-terminus [8], [10]. Together with Ewing’s sarcoma (EWS) and RNA polymerase II, TATA box binding protein (TBP)-associated factor, 68 kDa (TAFII68/TAF15), FUS belongs to a family called TET or FET. Since the vast majority of the ALS mutations occur in the NLS (amino acids 514–526) and result in cytoplasmic retention of FUS protein, mutations could impair its function or lead to a toxic gain-of-function [11], [12]. Even though mutations in *FUS* account for only a small fraction of FALS and SALS patients, it has been suggested that FUS protein may be a common component of cellular inclusions in non-SOD1 ALS and other neurodegenerative conditions [13]. Cytosolic mislocalization of FUS has already been shown to kindle misfolding of wild-type SOD1 in non-SOD1 ALS, implying a shared pathogenic pathway underlying SALS, non-SOD1 FALS, ALS/FTD, and related disorders [13], [14].

Given the role of FUS in RNA processing, it could be hypothesized that mutant FUS contributes to ALS by altering expression of many genes. A very recent study did identify more than 5,500 RNA targets of FUS in mouse and human brain, and showed that depletion of FUS changed at least 600 mRNA levels and 350 splicing patterns [15]. However, the influence of overexpressed wild-type and mutant forms of *FUS* on global expression has yet to be determined. Towards this goal, we have performed RNA-Seq on cells expressing exogenous wild-type *FUS*, two mutant forms of *FUS* (R521G, R522G) or small interfering RNA (siRNA) against *FUS*. The results of this study yield insights into the normal pathways influenced by wild-type FUS and how mutations lead to the pathogenesis of ALS.

## Materials and Methods

### Plasmid Constructs

Human wild-type *FUS* (clone MGC-8537, Invitrogen, Carlsbad, CA) was inserted into a pcDNA3.1 vector containing an N-terminal V5 (Invitrogen) epitope tag with BP and LR Clonase kits (Invitrogen). Mutations located in exon 15 (p.Arg521Gly (R521G [c.C1561G]) and p.Arg522Gly (R522G [c.C1564G])) were generated by using QuikChange II Site-Directed Mutagenesis kit (Stratagene, La Jolla, CA). Sequencing was used to verify the orientation of the inserts and absence of polymerase chain reaction (PCR)-induced mutations.

### Cell Culture

HEK-293 cells optimized for transfections (HEK-293T) were cultured in Dulbecco’s Modified Eagle’s medium (DMEM) supplemented with 10% fetal calf serum and 4 mM L-glutamine. Transfections were performed with Lipofectamine 2000 transfection reagent (Invitrogen) according to the manufacturer’s recommendations. Cells were transfected with either 4 µg of expression constructs or 50.0 ρmol *Silencer* pre-designed siRNA directed against *FUS* (s5402, Applied Biosystems, Carlsbad, CA). To test the efficiency of transfections, cells were co-transfected with 0.4 µg enhanced green fluorescent protein (pEGFP-C1) plasmid, which expressed GFP (Clontech, Mountain View, CA). After 24 hours the medium was changed to DMEM and cells were analyzed at 48 hours post-transfection. Transfection efficiencies for all conditions were greater than 75%, as determined by immunofluorescence staining.

### Preparation of Cell Lysates

Transfected cells were washed in phosphate-buffered saline (PBS) and then detached with a cell scraper. An aliquot of the cell suspension was centrifuged, resuspended in TENN buffer (50 mM Tris-HCl [pH 7.4], 5 mM EDTA, 150 mM NaCl, 0.5% Nonidet P-40) with Protease Inhibitor Cocktail (Roche, Basel, Switzerland) and analyzed by western blot. Western blot analysis was performed using the following antibodies: rabbit anti-FUS (1∶5,000, Bethyl Laboratories, Montgomery, TX, A300-302A), mouse anti-V5 (1∶5,000, Invitrogen, catalog # 37-7500), rabbit anti-glyceraldehyde 3-phosphate dehydrogenase (GAPDH, 1∶15,000; Abcam, Cambridge, UK, catalog # Ab22555), Odyssey IRDye anti-rabbit IgG (1∶20,000) and Odyssey IRDye anti-mouse IgG (1∶20,000). The Odyssey Infrared Imaging System (Li-cor, Lincoln, NE) was used for quantification of western blots. RNA was isolated from the remaining cell suspension according to the RNeasy Mini Kit protocol (Qiagen, Hilden, Germany).

### RNA-Seq and Data Analysis

RNA-Seq libraries were generated as described previously [16]. Briefly, polyA+ RNA was reverse transcribed using Superscript III and converted to double-stranded cDNA. Illumina adapters were ligated and libraries were amplified by PCR and size selected by gel electrophoresis prior to sequencing on an Illumina Genome Analyzer (Illumina, San Diego, CA). Analysis of RNA-Seq was performed utilizing software package ExpressionPlot [17]: gene expression levels were estimated by counting the number of reads mapping to constitutive exons for each gene and determining RPKM values (reads per kilobase of exon model per million uniquely mapped reads). P-values were calculated using the Fisher’s exact test for pairwise comparison between samples, which is shown to be conservative, as described elsewhere [17]. For skipped exon analysis, P-values were determined based on the ratio of inclusion reads to the sum of skipping and flanking reads (Materials and Methods S1 in File S1) using Fisher’s exact test. For intron retention analysis, P-values were calculated using the Fisher’s exact test based on the comparison of inclusion and flanking reads between conditions (Materials and Methods S1 in File S1). Plots demonstrating the distribution of data by showing the log intensity ratio (M) and the average log intensity for a dot (A, [MA plots]) per condition comparison did not display expression level dependent differential signals (Figure S1 in File S1).

Events with Ensemble gene identifiers were subjected to further analysis. For the differential expression analysis, events without RPKM units were excluded. After Bonferroni multiple test correction, significant events (P-value <0.05) were selected and used to perform functional annotation and functional domain analysis by Kyoto Encyclopedia of Genes and Genomes (KEGG) pathway analysis. Details of all significant events can be found in the Supplementary spreadsheet in File S2. To avoid length-dependent bias [18], two different background lists were initially utilized: the *Homo sapiens* background list as supplied by Database for Annotation, Visualization and Integrated Discovery (DAVID) Functional Annotation Tool (http://david.abcc.ncifcrf.gov/home.jsp) and the list generated from cells transfected with the vector alone. However, the results from the two approaches displayed few differences. Therefore, only analysis using the *Homo sapiens* background list is reported for all conditions. Venn diagrams were generated comparing the vector condition to wild-type FUS, the two FUS mutants and siRNA against *FUS*, using an online tool (http://bioinfogp.cnb.csic.es/tools/venny/index.html). Our data has been deposited to the ExpressionPlot Web Server (http://als-research.dyndns-server.com/cgi-bin/expressionplot/home.pl) [17], and will be accessible from the date of publication.

### PCR

To confirm gene expression levels detected by RNA-Seq, we performed quantitative real-time PCR (qRT-PCR) with SYBR Green PCR mix (Qiagen), according to the manufactures guidelines. Expression was compared between our conditions (wild-type FUS, siRNA, mutants and vector alone; the same samples were used for RT-PCR and RNA-Seq). Six differentially expressed genes displaying at least one comparison with a minimal log_2_ fold change in expression of 0.4 (>1.32 fold change) were chosen for confirmation by RT-PCR (File S2). The following PCR program was used: denaturation at 95°C for five minutes followed by 35 cycles of denaturation at 95°C, annealing at 58/60°C, and extension at 72°C, each for 30 seconds. Primer pairs used for this reaction are as follows: (1) RNA binding motif protein 25 (*RBM25*) ATGAGCATTATGGCTCCTGCTCCA/TGCTTTCCATTCATCCAGCTGTGC, (2) *TAF15* TCCTTCAGCTAAGGCAGCCATTGA/GGCTCATTGCACTGATTGCAGGAA, (3) threonyl-tRNA synthetase (*TARS*) TTTGAGGATGAGGAAGCTCAGGCA/TTGCCCGTGTGTCTAACATGAGGA, (4) translocated promoter region, nuclear basket protein (*TPR*) AACAACTCCGCAAATCACGACAGC/TGTTTAAGGGCAGCCTTAGCCTCT, (5) amyloid beta (A4) precursor protein (*APP*) ACCAACCAGTGACCATCCAGAACT/CAGCAACATGCCGTAGTCATGCAA, and (6) hematological and neurological expressed 1 (*HN1*) TGGGTTTACCAAGCCTCAACTGGA/AGGAAGACCCGCTTCAGTGTGATT. For differential expression analysis, threshold cycle (Ct) values were collected in quadruplicate for each condition, and delta-Ct values were normalized using GAPDH values for the same condition. Hereafter, the average Ct value for the reference condition was subtracted from the normalized Ct values (delta-delta-Ct) and the fold decrease change was calculated (&2circ;(delta-delta-Ct)). Subsequently, the relative expression and log_2_ fold changes were determined.

For the confirmation of alternative splicing RNA-Seq results, semi-quantitative PCR was performed using primers derived from the upstream and downstream exons. Two genes (*PRPF8* and *RPS24*) displaying a minimal log_2_ fold change in exon exclusion:inclusion of 0.4 (>1.32 fold change) were chosen for confirmation. Quadruplicate reverse transcribed PCR products were separated on a 2% gel, and bands were analyzed with ImageJ (http://rsbweb.nih.gov/ij/). For each condition, an unspliced:spliced ratio and log_2_ fold change were calculated. Primer pairs used for this reaction are as follows: (1) pre-mRNA-processing-splicing factor 8 (*PRPF8*) TTGGGAATCTGGTTCAGTCC/GGCACACACTGGCTTATGAT, and (2) ribosomal protein S24 (*RPS24*) ATGAAGAAAGTCAGGGGGACTG/TAATGTTCTTGCGAAAAATCCAC. A general linear model was fitted with delta-delta-Ct values or unspliced:spliced values as dependent variable, and all conditions as categorical independent variable, using the appropriate condition as reference (e.g. wild-type FUS, when comparing one mutant with wild-type FUS).

## Results

To investigate the global effects of wild-type and mutant FUS protein on cellular transcription and splicing, HEK-293T cells were transfected with expression vectors encoding wild-type FUS, two FUS mutants (R521G and R522G) or siRNA directed against *FUS*. Transfection with an empty vector served as a control condition. RNA-Seq resulted in 24 to 31 million reads for each transfection condition (Table S1 in File S1). Quality control assessments revealed that >69.9% of reads uniquely mapped to the genome and less than 2.72% of reads were derived from ribosomal RNA (Table S1 in File S1). Analysis of RNA-Seq data demonstrated that cells expressing either wild-type or mutant *FUS* displayed a ∼2-fold increase in expression, whereas cells harboring siRNA displayed a ∼4-fold decrease in *FUS* expression (Figure S2 in File S1). Interestingly, western blot analysis of an aliquot of transfected cells used for RNA-Seq displayed lower levels of overexpression of *FUS* mutants relative to the wild-type, suggesting that post-transcriptional regulation may influence FUS protein levels (Figure S3 in File S1).

Pathway analysis was performed for differential expressed genes using the DAVID Functional Annotation Tool (Materials and Methods). This analysis revealed that overexpression of wild-type *FUS* (wild-type FUS vs. vector alone) influences expression of ribosomal related genes, whereas knock-down of *FUS* expression (siRNA vs. vector alone) influences expression of both ribosome and spliceosome related genes (Table 1, Table S2 in File S1). The differentially expressed genes encoded several ribosomal proteins, aspartic acid, glutamic acid, alanine, and aspartic acid (DEAD) box proteins, RNA-binding motif proteins, hnRNPs, and splicing factors (Table S3 in File S1). Further analysis revealed that the differentially expressed genes often encoded proteins containing RRMs, endoplasmic reticulum (ER) targeting sequences, and nucleotide-binding alpha-beta plaits (Table S4 in File S1).

**Table 1:** Functional pathway analysis of differentially expressed genes.

Group	KEGG Pathway	Count	P-value	Benjamini and Hochberg, FDR, P-value
**Wild-type vs. vector**	Ribosome	14	5.1E-5	6.6E-3
**siRNA vs. vector**	Spliceosome	34	1.4E-8	2.2E-6
	Ribosome	26	1.2E-7	1.0E-5
**R521G vs. vector**	Spliceosome	18	1.0E-8	9.2E-7
**R522G vs. vector**	Ribosome	15	4.0E-7	4.0E-5
	Spliceosome	15	3.5E-5	1.8E-3
	Mismatch repair	6	8.0E-4	2.7E-2
	DNA replication	7	9.1E-4	2.3E-2

FDR = False Discovery Rate.

To understand the mechanism by which FUS mutants may contribute to ALS pathogenesis, we determined whether the expression patterns induced by mutant FUS were more similar to reduced *FUS* expression or overexpressed wild-type *FUS*. Similarity to reduced expression of *FUS* would suggest that the mutants act by a loss-of-function mechanism, whereas similarity to overexpressed wild-type *FUS* would suggest a gain-of-function for the mutants. Towards this end, the number of differentially expressed genes between each of the two FUS mutants and either siRNA or wild-type FUS conditions was calculated. As shown in Table 2, both the R521G and R522G mutants displayed an increased number of differentially expressed genes when compared to reduced *FUS* expression than to overexpressed wild-type conditions (P-value <0.0001 for both). In other words, the transcriptional profiles of cells expressing this *FUS* mutant more closely resembled that of overexpressed wild-type *FUS* than of reduced levels of *FUS*. These results suggested that the FUS mutants do not contribute to ALS pathogenesis through a loss-of-function.

**Table 2:** Number of differentially expressed genes by mutant FUS, *FUS* overexpression, and *FUS* knock-down.

		Wild-type (n)	siRNA (n)	Fisher’s exact, P-value
**R521G**	Significant	566	863	5.4E-17
	Non-significant	17,122	16,511	
**R522G**	Significant	1,198	1,937	7.4E-47
	Non-significant	16,610	15,583	
**R521G**	Up-regulated	509	423	1.3E-62
	Down-regulated	57	440	
**R522G**	Up-regulated	112	88	1.6E-07
	Down-regulated	1,086	1,849	

To investigate whether the two FUS mutants differed in their influence on transcription, the direction of change for differentially expressed genes was compared. Interestingly, although the total number of genes that was regulated by R521G and R522G was not significantly different, there was a significant difference in the number of genes that was up/down-regulated. As shown in Table 3, R521G dramatically favored up-regulation of the differentially identified genes, whereas R522G favored down-regulation. Despite the fact that the two FUS mutants appeared to have opposite effects on differential gene expression, pathway analysis revealed that both mutants significantly influenced spliceosome related genes (Table 1). Additionally, the R522G mutant also altered ribosome, mismatch repair and DNA replication related genes. The identified genes encoded, amongst others, DEAD box proteins, hnRNPs, splicing factors, ribosomal proteins, exonuclease, replication factors, mini-chromosome maintenance complex components, and proliferating cell nuclear antigen (Table S3 in File S1). The most significantly enriched protein domains were RRMs, nucleotide-binding alpha-beta plaits and helicases (Table S4 in File S1).

**Table 3:** The FUS R521G mutant causes increased up-regulation of genes as compared to the R522G mutant.

		R521G (n)	R522G (n)	Fisher’s exact,P-value
**Vector**	Significant	332	328	0.78
	Non-significant	17,052	17,214	
**Vector**	Increased expression	297	8	3.7E-134
	Decreased expression	35	320	

**Figure 1:**
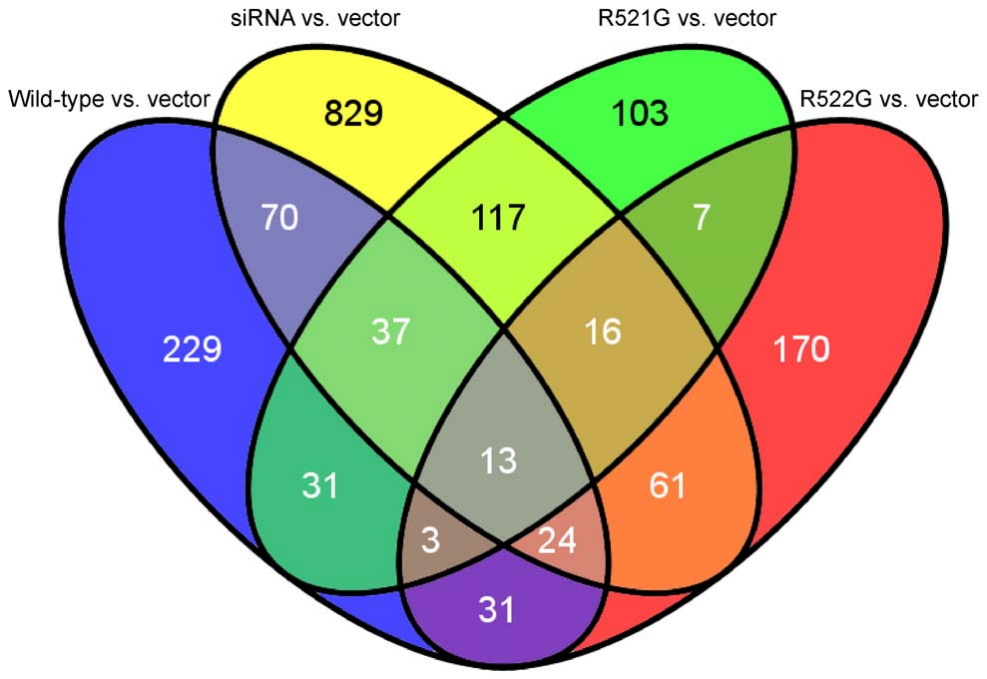
Venn diagram of differentially expressed genes. A comparison between each condition (wild-type FUS, siRNA against *FUS* and mutants) and the vector reveals that 13 differentially expressed genes are shared amongst them (Table 4).

Venn diagrams were generated (Figure 1) to identify genes shared amongst conditions. When both mutants, wild-type FUS and siRNA, directed against *FUS,* were compared to the vector condition, 13 genes were revealed (Table 4), including *HN1*, ribosomal protein S3, S16, S19 (*RPS3*, *RPS16*, *RPS19*), CCR4-NOT transcription complex, subunit 1 (*CNOT1*), and RNA component of mitochondrial RNA processing endoribonuclease (*RMRP*).

**Table 4:** Genes shared amongst all conditions for differential expression analysis and skipped exon analysis as shown by Venn diagrams.

	Gene	Description
**Differential expression**	HN1	hematological and neurological expressed 1
	RPS3	ribosomal protein S3
	HSPA5	heat shock 70 kDa protein 5
	RPS16	ribosomal protein S16
	RPS19	ribosomal protein S19
	FLNA	filamin A, alpha, actin-binding protein
	CNOT1	CCR4-NOT transcription complex, subunit 1, transcription repressor
	CALM3	calmodulin 3 (phosphorylase kinase, delta)
	NFKB2	nuclear factor of kappa light polypeptide gene enhancer in B-cells 2 (p49/p100)
	PLK1	polo-like kinase 1, serine/threonine-protein kinase
	RMRP	RNA component of mitochondrial RNA processing endoribonuclease
	EIF4G2	eukaryotic translation initiation factor 4 gamma, 2
	NCL	nucleolin, synthesis and maturation of ribosomes
**Exon skipping**	RPL34	ribosomal protein L34
	PRKDC	protein kinase, DNA-activated, catalytic polypeptide

To assess the reliability of our RNA-Seq differential expression analysis, we attempted to validate a subset by qRT-PCR. Towards this end, we selected 10 significant expression differences from 6 different genes observed by our RNA-Seq results (*RBM25*, *TAF15*, *TARS*, *TPR*, *APP*, and *HN1*) for validation. Through our analysis, we were able to validate 8 out of the 10 observed expression changes by qRT-PCR (Figure S4 in File S1), suggesting that our RNA-Seq results are reliable.

Our analysis was extended to investigate the influence on alternative splicing, in particular skipped exons. Comparison of overexpressed wild-type *FUS* to vector transfected cells resulted in only 32 significant events and did not reveal any functional pathways enriched by these events (Table S2 in File S1). In contrast, knock-down of *FUS* expression resulted in 579 significant changes in skipped exons splicing patterns. These changes were enriched in ribosome and spliceosome related genes (Table S2 in File S1). Pathway analysis of the R521G mutant revealed involvement of ribosome related genes, whereas the R522G mutant also affected spliceosome related genes (Table S2 in File S1). Additionally, we detected a small significant difference in the number of genes that were affected by R521G and R522G mutations, but there was no significant difference in the direction of this change (Table 5). Semi-quantitative PCR of two alternatively spliced genes, *PRPF8* and *RPS24*, demonstrated similar splicing patterns by both techniques (Figure S5 in File S1).

**Table 5:** The FUS R521G mutant induces increased skipping of exons relative to the R522G mutant.

		R521G (n)	R522G (n)	Fisher’s exact,P-value
**Vector**	Significant	106	64	0.0031
	Non-significant	65,812	63,215	
**Vector**	Increased skipping	42	30	0.35
	Decreased skipping	64	34	

To further investigate the influence of FUS on alternative splicing, we similarly analyzed our results for genes displaying changes in retained introns. Overexpression of wild-type *FUS* revealed 3,116 significant retained intron events. Pathway analysis showed these event were enriched in spliceosome, Huntington’s disease, proteasome, Parkinson’s disease, oxidative phosphorylation, cell cycle, DNA replication, and pyrimidine metabolism related genes (Table S2 in File S1). Down-regulation of *FUS* resulted in 3,054 significant retained intron events that were involved in the same pathways. There was a significant difference in the number of genes that were affected by R521G and R522G (P-value 5.2E-08). The direction of this change was significantly different as well: the R522G mutant displayed increased intron retention relative to the R521G mutant (Table 6). Pathway analysis revealed enrichment for genes related to the spliceosome, Huntington’s disease, proteasome, DNA replication, cell cycle, pyrimidine metabolism oxidative phosphorylation, and RNA polymerase (Table S2 in File S1).

**Table 6:** The FUS R522G mutant induces increased retention of introns relative to the R521G mutant.

		R521G (n)	R522G (n)	Fisher’s exact,P-value
**Vector**	Significant	2,932	2,521	5.2E-08
	Non-significant	109,789	109,655	
**Vector**	Increased retention	621	1,086	6.3E-68
	Decreased retention	2,311	1,435	

**Figure 2:**
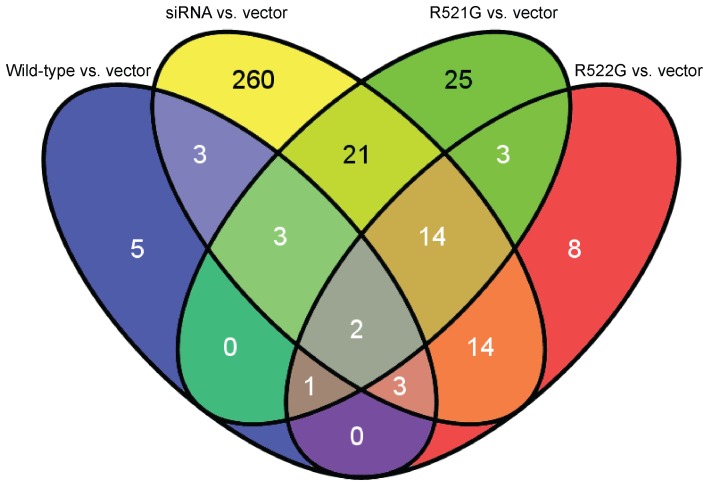
Venn diagram of genes demonstrating exon skipping. Two overlapping genes are identified when significant splicing events are compared between conditions (Table 4).

**Figure 3:**
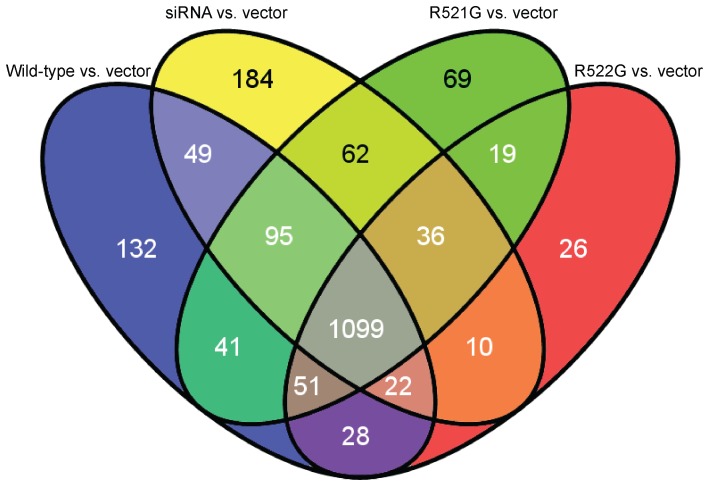
Venn diagram of genes displaying intron retention. A Venn diagram that compares our conditions to the vector shows that 1,099 retained intron events are shared (Table 7 and Table S5 in File S1).

We created Venn diagrams to determine which genes were shared amongst all conditions for both alternative splicing analyses. A Venn diagram of skipped exon analysis identified two genes (Figure 2): ribosomal protein L34 (*RPL34*) and protein kinase, DNA-activated, catalytic polypeptide (*PRKDC*). Retained intron analysis identified 1,099 genes (Figure 3); pathway analysis of these genes resulted in 15 significant KEGG pathways (Table 7). The most significant pathways were related to the spliceosome, Huntington’s disease, DNA replication, the proteasome, and pyrimidine metabolism (Table S5 in File S1).

**Table 7:** Functional pathway analysis of shared genes for retained introns analysis as shown by Venn diagram.

KEGG Pathway	Count	P-value	Benjamini and Hochberg, FDR, P-value
Spliceosome	43	1.9E-16	3.6E-14
Huntington’s disease	42	5.6E-10	4.5E-8
DNA replication	16	4.0E-8	2.2E-6
Proteasome	18	6.1E-8	2.5E-6
Pyrimidine metabolism	25	3.2E-7	1.0E-5
RNA polymerase	12	5.5E-6	1.5E-4
Cell cycle	27	5.6E-6	1.3E-4
Parkinson’s disease	27	8.9E-6	1.8E-4
Purine metabolism	30	1.1E-5	2.0E-4
Aminoacyl-tRNA biosynthesis	14	1.2E-5	2.0E-4
Oxidative phosphorylation	25	1.0E-4	1.5E-3
Alzheimer’s disease	28	2.5E-4	3.4E-3
Nucleotide excision repair	12	5.9E-4	7.4E-3
Mismatch repair	8	1.8E-3	2.0E-2
Citrate cycle (TCA cycle)	9	2.7E-3	2.8E-2

## Discussion

To study the effects of wild-type and mutant forms of FUS, we utilized RNA-Seq analysis to investigate alterations in gene expression and alternative splicing. Our results show that wild-type FUS affects ribosomal and spliceosome related genes, and that proteins containing RRMs and nucleotide-binding alpha-beta plaits were most frequently influenced, confirming the important role of FUS in RNA processing pathways [8]. This role is strengthened by previous reports; it has already been shown that FUS associates with products of RNA polymerase II transcription, forms complexes with hnRNPs, and represses RNA polymerase III transcription [19]–[21]. Furthermore, FUS inhibits the acetyltransferase activities of CREB-binding protein (CBP) and p300 on cyclin D1 (CCND1) [22], and regulates the transcription factor nuclear factor kB (NF-kB) [23], [24]. FUS also engages in rapid nucleocytoplasmic shuttling [25], associates with actin-dependent motor protein myosin Va (MyoVa) [26], [27], and is a component of RNA granules that transport mRNAs [28]. Splicing factors, such as serine and arginine (SR) proteins, form complexes with FUS and removal of FUS from the nuclear extract causes disturbances of the splicing factor equilibrium [29]. Additionally, photoactivatable ribonucleoside-enhanced cross-linking and immunoprecipitation (PAR-CLIP) analysis has shown that FUS binds RNA at high frequency, and preferably near splice acceptors [30]. Very recently, high-throughput sequencing and computational approaches demonstrated FUS binding sites in thousands of mouse and human brain pre-mRNAs [15]. Moreover, in mice, FUS depletion with single-stranded antisense oligonucleotides (ASOs) resulted in up-regulation of 275 genes, down-regulation of 355 genes, and 374 splicing events [15].

FUS shows several structural and functional similarities with transactive response DNA-binding protein 43 (TDP-43) [31]. Mutations in *TARDBP*, which encodes TDP-43, have been identified in ∼5% of ALS patients [32]. Similar to FUS, the role of TDP-43 is diverse and includes transcriptional regulation, splicing inhibition, regulation of mRNA transport, repression of translation, and mRNA degradation [8]. Recently, it has been shown that TDP-43 interacts with a diverse spectrum of RNAs with important functions in the brain [33]. Depletion of TDP-43 from mouse adult brain resulted in a reduction of long introns, which encode proteins involved in synaptic activity [34]. Deep sequencing further identified more than 4,300 TDP-43 RNA-binding partners in rat cortical neurons [35]. These RNA partners were particularly enriched for genes related to synaptic function, RNA metabolism, and neuronal development [35]. Thus, RNA targets of both FUS and TDP-43 emphasize that alterations in RNA processing pathways play a central role in neurodegenerative diseases [31], [32].

In the present study, we have revealed that FUS mutants are more similar to overexpression of wild-type *FUS* than to our knock-down condition. These results suggest that mutants do not contribute to ALS pathogenesis through a loss-of-function, and are supported by recent findings. *Caenorhabditis elegans* expressing mutant *FUS*, for instance, demonstrated adult-onset, age-dependent loss of motility, progressive paralysis and neuronal degeneration [36]; although mutant phenotypes could be rescued by methylene blue [37], this could not be established by overexpressing wild-type *FUS* [38]. These findings are further substantiated by reports that transgenic mice overexpressing wild-type *FUS* developed an aggressive phenotype with limb paralysis and death by twelve weeks in homozygous animals [39], and that *FUS* mutations in *Drosophila* appeared to cause adult-onset neurodegeneration via a gain-of-toxicity [40]. Whether mutations in *FUS* are indeed causative for ALS through a gain-of-function mechanism is, however, still a matter of debate, especially since other studies have advocated a loss-of-function mechanism [15], [32], [41]–[43].

Our analysis also identified differences between R521G and R522G FUS mutants. Differential expression analysis demonstrated that these mutations appeared to have opposite effects on transcriptional regulation. Moreover, both mutations influenced the spliceosome, but the R522G mutation also altered genes involved in ribosomal processes, mismatch repair, and DNA replication. In addition, analysis of skipped exons revealed that both FUS mutants affected ribosomal genes; the R522G mutant influenced spliceosome related genes as well. Retained intron analysis displayed that intron retention was more frequently detected for R522G than for R521G, suggesting that R522G mutations cause more profound changes than R521G mutations. Previously, R521G and R522G mutations have been studied in several experimental models. Yeast strains have been developed expressing wild-type and mutant *FUS* [44]–[47]. Overexpression of both resulted in punctuate aggregates in the cytoplasm; R521G aggregated with very similar kinetics to wild-type FUS in protein aggregation assays [44]–[47]. *In vitro* studies, demonstrated that the R521G mutant caused a relatively mild cytoplasmic mislocalization, whereas R522G caused a strong mislocalization [48]. The R522G mutant was also investigated in neuroblastoma cells, and was shown to predominantly accumulate in the cytoplasm and formed aggregates varying in size and shape [49]. In *Caenorhabditis elegans,* the motor function and lifespan of animals expressing R521G mutations was indistinguishable from wild-type FUS, whereas R522G mutations caused a significant decrease in motor function and lifespan [38]. These observations suggest that FUS mutants may act through differing but converging mechanisms leading to ALS.

Researchers planning on pursuing differential expression or altered splicing observations from any transcriptome-wide study should always confirm results through an alternative method. Here, we have attempted to address the reliability of our RNA-Seq data through validation by RT-PCR (differential expression) and semi-quantitative PCR (exon skipping). Among the specific molecular targets that were affected by FUS, we validated the expression patterns of *RBM25*, *TAF15*, *TARS*, *TPR*, and *APP*, and the alternative splicing of *PRPF8* and *RPS24*. In the differential expression analysis, as well as the skipped exons and retained introns analyses, proteins with spliceosomal and ribosomal functions were among the most prominent molecular targets. These results underscore the function of FUS in the translation of mRNA to proteins, and splicing of introns from pre-mRNAs, respectively.

In addition to caveats of analyzing RNA-Seq data on an individual gene level, we have additionally performed KEGG pathway analysis. This methodology is highly dependent on the selection of a proper reference gene list. There is extensive literature on random lists of genes that appear to be “enriched” for specific pathways [50], which emphasizes the importance of using an appropriate reference list. Many studies use a specific platform to acquire data, which, by definition, could enrich itself. For RNA-Seq data a potential length-dependent bias has to be considered as well [18]. We addressed this issue by using both our successfully sequenced transcripts and the *Homo sapiens* background list from DAVID as reference lists; this minimized bias, although we cannot completely exclude the possibility of residual bias. Furthermore, technical limitations hampered our ability to detect splicing events, for instance, our reads were relatively short as compared to the improved methods that are currently available.

To summarize, we have shown that FUS is implicated in the regulation of ribosomal and spliceosome related genes, highlighting the importance of RNA processing pathways in the pathogenesis of ALS. Furthermore, the expression changes induced by FUS mutants suggest that they do not contribute to ALS pathogenesis through a loss-of-function. Finally, our results demonstrate that R521G and R522G mutations display differences in their influence on transcription and splicing. Taken together, these observations provide additional insights into the normal function of FUS and how mutations lead to the development of ALS.

## Supporting Information

File S1 **This file includes supporting materials and methods, Tables S1–S5, and Figures S1–S5.** Legends for Figure S1–S5 are as follows: **Figure S1. MA plots per condition comparison.** MA plots per comparison where M = log (rpkm_condition_) – log (rpkm_vector_) and A = 0.5 * (log (rpkm_condition_)+log (rpkm_vector_)). **Figure S2.** ***FUS*** **gene expression levels measured by RNA-Seq.** Reads per kilobase of exon model per million uniquely mapped reads (RPKM) counts are displayed for each condition. Transfections with wild-type *FUS*, R521G and R522G resulted in a ∼2-fold increase in expression level, transfections with siRNA in a ∼4-fold decrease in expression level. **Figure S3. FUS protein levels measured by western blot.** The same samples were used for western blotting and RNA-Seq. We stained for FUS and the V5-tag (Materials and Methods), and bands were subsequently detected and quantified with an Odyssey Infrared Imaging System. For FUS, we compared each condition to the vector and calculated relative integrated intensities. We demonstrated that siRNA caused a decrease in *FUS* expression (1.0 versus 0.2), whereas transfections with wild-type *FUS* and the two mutants caused an increase in *FUS* expression (1.0 versus 4.2, 1.8 and 1.1). The multiple bands detected by FUS antibodies have been observed previously [51]. **Figure S4. Correlation of RNA-Seq and RT-PCR for** ***RBM25*** **,** ***TAF15*** **,** ***TARS*** **,** ***TPR*** **,** ***APP*** **and** ***HN1*** **.** We selected 10 differentially expressed conditions within six genes for RT-PCR validation. These genes required at least one comparison with a log_2_ fold change of 0.4 (1.32 fold change). For each comparison, the log_2_ fold change and corresponding P-values are shown. Validation was observed in 8 out of 10 comparisons for 5 out of 6 genes. **Figure S5. Correlation of RNA-Seq and Semi-quantitative PCR for** ***PRPF8*** **and** ***RPS24*** **.** Two alternatively spliced genes, pre-mRNA-processing-splicing factor 8 (*PRPF8*) and ribosomal protein S24 (*RPS24*), with a log_2_ fold change in exon exclusion:inclusion above 0.4, (1.32 fold-change) were selected for RT-PCR. The log_2_ fold change and corresponding P-values are shown. RNA-Seq results were validated by semi-quantitative PCR in both cases.
(PDF)

File S2 **This Excel spreadsheet file contains detail of all significant events observed from the RNA-Seq analysis.**
(XLS)

## References

[1] Rowland LP, Shneider NA (2001) Amyotrophic lateral sclerosis. N Engl J Med 344: 1688–1700. 11386269 10.1056/NEJM200105313442207

[2] Andersen PM, Al-Chalabi A (2011) Clinical genetics of amyotrophic lateral sclerosis: what do we really know? Nat Rev Neurol 7: 603–615. 21989245 10.1038/nrneurol.2011.150

[3] van Blitterswijk M, van Es MA, Hennekam EA, Dooijes D, van Rheenen W, et al (2012) Evidence for an oligogenic basis of amyotrophic lateral sclerosis. Hum Mol Genet 21: 3776–3784. 22645277 10.1093/hmg/dds199

[4] DeJesus-Hernandez M, Mackenzie IR, Boeve BF, Boxer AL, Baker M, et al (2011) Expanded GGGGCC hexanucleotide repeat in noncoding region of C9ORF72 causes chromosome 9p-linked FTD and ALS. Neuron 72: 245–256. 21944778 10.1016/j.neuron.2011.09.011 PMC3202986

[5] Renton AE, Majounie E, Waite A, Simon-Sanchez J, Rollinson S, et al (2011) A hexanucleotide repeat expansion in C9ORF72 is the cause of chromosome 9p21-linked ALS-FTD. Neuron 72: 257–268. 21944779 10.1016/j.neuron.2011.09.010 PMC3200438

[6] Wu CH, Fallini C, Ticozzi N, Keagle PJ, Sapp PC, et al (2012) Mutations in the profilin 1 gene cause familial amyotrophic lateral sclerosis. Nature 488: 499–503. 22801503 10.1038/nature11280 PMC3575525

[7] Mackenzie IR, Rademakers R, Neumann M (2010) TDP-43 and FUS in amyotrophic lateral sclerosis and frontotemporal dementia. Lancet Neurol 9: 995–1007. 20864052 10.1016/S1474-4422(10)70195-2

[8] van Blitterswijk M, Landers JE (2010) RNA processing pathways in amyotrophic lateral sclerosis. Neurogenetics 11: 275–290. 20349096 10.1007/s10048-010-0239-4

[9] Calvio C, Neubauer G, Mann M, Lamond AI (1995) Identification of hnRNP P2 as TLS/FUS using electrospray mass spectrometry. RNA 1: 724–733. 7585257 PMC1369314

[10] Gal J, Zhang J, Kwinter DM, Zhai J, Jia H, et al.. (2011) Nuclear localization sequence of FUS and induction of stress granules by ALS mutants. Neurobiol Aging 32: 2323 e2327–2340. 10.1016/j.neurobiolaging.2010.06.010 PMC2997923 20674093

[11] Kwiatkowski TJ Jr, Bosco DA, Leclerc AL, Tamrazian E, Vanderburg CR, et al (2009) Mutations in the FUS/TLS gene on chromosome 16 cause familial amyotrophic lateral sclerosis. Science 323: 1205–1208. 19251627 10.1126/science.1166066

[12] Vance C, Rogelj B, Hortobagyi T, De Vos KJ, Nishimura AL, et al (2009) Mutations in FUS, an RNA processing protein, cause familial amyotrophic lateral sclerosis type 6. Science 323: 1208–1211. 19251628 10.1126/science.1165942 PMC4516382

[13] Deng HX, Zhai H, Bigio EH, Yan J, Fecto F, et al (2010) FUS-immunoreactive inclusions are a common feature in sporadic and non-SOD1 familial amyotrophic lateral sclerosis. Ann Neurol 67: 739–748. 20517935 10.1002/ana.22051 PMC4376270

[14] Pokrishevsky E, Grad LI, Yousefi M, Wang J, Mackenzie IR, et al (2012) Aberrant localization of FUS and TDP43 is associated with misfolding of SOD1 in amyotrophic lateral sclerosis. PLoS One 7: e35050. 22493728 10.1371/journal.pone.0035050 PMC3320864

[15] Lagier-Tourenne C, Polymenidou M, Hutt KR, Vu AQ, Baughn M, et al.. (2012) Divergent roles of ALS-linked proteins FUS/TLS and TDP-43 intersect in processing long pre-mRNAs. Nat Neurosci. 10.1038/nn.3230 PMC3586380 23023293

[16] Wang ET, Sandberg R, Luo S, Khrebtukova I, Zhang L, et al (2008) Alternative isoform regulation in human tissue transcriptomes. Nature 456: 470–476. 18978772 10.1038/nature07509 PMC2593745

[17] Friedman BA, Maniatis T (2011) ExpressionPlot: a web-based framework for analysis of RNA-Seq and microarray gene expression data. Genome Biol 12: R69. 21797991 10.1186/gb-2011-12-7-r69 PMC3218831

[18] Oshlack A, Wakefield MJ (2009) Transcript length bias in RNA-seq data confounds systems biology. Biol Direct 4: 14. 19371405 10.1186/1745-6150-4-14 PMC2678084

[19] Zinszner H, Albalat R, Ron D (1994) A novel effector domain from the RNA-binding protein TLS or EWS is required for oncogenic transformation by CHOP. Genes Dev 8: 2513–2526. 7958914 10.1101/gad.8.21.2513

[20] Bertolotti A, Lutz Y, Heard DJ, Chambon P, Tora L (1996) hTAF(II)68, a novel RNA/ssDNA-binding protein with homology to the pro-oncoproteins TLS/FUS and EWS is associated with both TFIID and RNA polymerase II. EMBO J 15: 5022–5031. 8890175 PMC452240

[21] Tan AY, Manley JL (2010) TLS inhibits RNA polymerase III transcription. Mol Cell Biol 30: 186–196. 19841068 10.1128/MCB.00884-09 PMC2798296

[22] Wang X, Arai S, Song X, Reichart D, Du K, et al (2008) Induced ncRNAs allosterically modify RNA-binding proteins in cis to inhibit transcription. Nature 454: 126–130. 18509338 10.1038/nature06992 PMC2823488

[23] Goransson M, Andersson MK, Forni C, Stahlberg A, Andersson C, et al (2009) The myxoid liposarcoma FUS-DDIT3 fusion oncoprotein deregulates NF-kappaB target genes by interaction with NFKBIZ. Oncogene 28: 270–278. 18850010 10.1038/onc.2008.378

[24] Uranishi H, Tetsuka T, Yamashita M, Asamitsu K, Shimizu M, et al (2001) Involvement of the pro-oncoprotein TLS (translocated in liposarcoma) in nuclear factor-kappa B p65-mediated transcription as a coactivator. J Biol Chem 276: 13395–13401. 11278855 10.1074/jbc.M011176200

[25] Zinszner H, Sok J, Immanuel D, Yin Y, Ron D (1997) TLS (FUS) binds RNA in vivo and engages in nucleo-cytoplasmic shuttling. J Cell Sci 110 (Pt 15): 1741–1750. 10.1242/jcs.110.15.1741 9264461

[26] Selamat W, Jamari I, Wang Y, Takumi T, Wong F, et al (2009) TLS interaction with NMDA R1 splice variant in retinal ganglion cell line RGC-5. Neurosci Lett 450: 163–166. 19103256 10.1016/j.neulet.2008.12.014

[27] Yoshimura A, Fujii R, Watanabe Y, Okabe S, Fukui K, et al (2006) Myosin-Va facilitates the accumulation of mRNA/protein complex in dendritic spines. Curr Biol 16: 2345–2351. 17141617 10.1016/j.cub.2006.10.024

[28] Belly A, Moreau-Gachelin F, Sadoul R, Goldberg Y (2005) Delocalization of the multifunctional RNA splicing factor TLS/FUS in hippocampal neurones: exclusion from the nucleus and accumulation in dendritic granules and spine heads. Neurosci Lett 379: 152–157. 15843054 10.1016/j.neulet.2004.12.071

[29] Meissner M, Lopato S, Gotzmann J, Sauermann G, Barta A (2003) Proto-oncoprotein TLS/FUS is associated to the nuclear matrix and complexed with splicing factors PTB, SRm160, and SR proteins. Exp Cell Res 283: 184–195. 12581738 10.1016/s0014-4827(02)00046-0

[30] Hoell JI, Larsson E, Runge S, Nusbaum JD, Duggimpudi S, et al (2011) RNA targets of wild-type and mutant FET family proteins. Nat Struct Mol Biol 18: 1428–1431. 22081015 10.1038/nsmb.2163 PMC3230689

[31] Lagier-Tourenne C, Polymenidou M, Cleveland DW (2010) TDP-43 and FUS/TLS: emerging roles in RNA processing and neurodegeneration. Hum Mol Genet 19: R46–64. 20400460 10.1093/hmg/ddq137 PMC3167692

[32] Da Cruz S, Cleveland DW (2011) Understanding the role of TDP-43 and FUS/TLS in ALS and beyond. Curr Opin Neurobiol 21: 904–919. 21813273 10.1016/j.conb.2011.05.029 PMC3228892

[33] Tollervey JR, Curk T, Rogelj B, Briese M, Cereda M, et al (2011) Characterizing the RNA targets and position-dependent splicing regulation by TDP-43. Nat Neurosci 14: 452–458. 21358640 10.1038/nn.2778 PMC3108889

[34] Polymenidou M, Lagier-Tourenne C, Hutt KR, Huelga SC, Moran J, et al (2011) Long pre-mRNA depletion and RNA missplicing contribute to neuronal vulnerability from loss of TDP-43. Nat Neurosci 14: 459–468. 21358643 10.1038/nn.2779 PMC3094729

[35] Sephton CF, Cenik C, Kucukural A, Dammer EB, Cenik B, et al (2011) Identification of neuronal RNA targets of TDP-43-containing ribonucleoprotein complexes. J Biol Chem 286: 1204–1215. 21051541 10.1074/jbc.M110.190884 PMC3020728

[36] Vaccaro A, Tauffenberger A, Aggad D, Rouleau G, Drapeau P, et al (2012) Mutant TDP-43 and FUS cause age-dependent paralysis and neurodegeneration in C. elegans. PLoS One 7: e31321. 22363618 10.1371/journal.pone.0031321 PMC3283630

[37] Vaccaro A, Patten SA, Ciura S, Maios C, Therrien M, et al (2012) Methylene Blue Protects against TDP-43 and FUS Neuronal Toxicity in C. elegans and D. rerio. PLoS One 7: e42117. 22848727 10.1371/journal.pone.0042117 PMC3407135

[38] Murakami T, Yang SP, Xie L, Kawano T, Fu D, et al (2012) ALS mutations in FUS cause neuronal dysfunction and death in Caenorhabditis elegans by a dominant gain-of-function mechanism. Hum Mol Genet 21: 1–9. 21949354 10.1093/hmg/ddr417 PMC3235006

[39] Mitchell JC, McGoldrick P, Vance C, Hortobagyi T, Sreedharan J, et al.. (2012) Overexpression of human wild-type FUS causes progressive motor neuron degeneration in an age- and dose-dependent fashion. Acta Neuropathol. 10.1007/s00401-012-1043-z PMC3549237 22961620

[40] Lanson NA Jr, Maltare A, King H, Smith R, Kim JH, et al (2011) A Drosophila model of FUS-related neurodegeneration reveals genetic interaction between FUS and TDP-43. Hum Mol Genet 20: 2510–2523. 21487023 10.1093/hmg/ddr150 PMC4288133

[41] Sasayama H, Shimamura M, Tokuda T, Azuma Y, Yoshida T, et al (2012) Knockdown of the Drosophila fused in sarcoma (FUS) homologue causes deficient locomotive behavior and shortening of motoneuron terminal branches. PLoS One 7: e39483. 22724023 10.1371/journal.pone.0039483 PMC3378546

[42] Wang JW, Brent JR, Tomlinson A, Shneider NA, McCabe BD (2011) The ALS-associated proteins FUS and TDP-43 function together to affect Drosophila locomotion and life span. J Clin Invest 121: 4118–4126. 21881207 10.1172/JCI57883 PMC3195475

[43] Halliday G, Bigio EH, Cairns NJ, Neumann M, Mackenzie IR, et al (2012) Mechanisms of disease in frontotemporal lobar degeneration: gain of function versus loss of function effects. Acta Neuropathol 124: 373–382. 22878865 10.1007/s00401-012-1030-4 PMC3445027

[44] Ju S, Tardiff DF, Han H, Divya K, Zhong Q, et al (2011) A yeast model of FUS/TLS-dependent cytotoxicity. PLoS Biol 9: e1001052. 21541368 10.1371/journal.pbio.1001052 PMC3082520

[45] Sun Z, Diaz Z, Fang X, Hart MP, Chesi A, et al (2011) Molecular determinants and genetic modifiers of aggregation and toxicity for the ALS disease protein FUS/TLS. PLoS Biol 9: e1000614. 21541367 10.1371/journal.pbio.1000614 PMC3082519

[46] Kryndushkin D, Wickner RB, Shewmaker F (2011) FUS/TLS forms cytoplasmic aggregates, inhibits cell growth and interacts with TDP-43 in a yeast model of amyotrophic lateral sclerosis. Protein Cell 2: 223–236. 21452073 10.1007/s13238-011-1525-0 PMC4875312

[47] Fushimi K, Long C, Jayaram N, Chen X, Li L, et al (2011) Expression of human FUS/TLS in yeast leads to protein aggregation and cytotoxicity, recapitulating key features of FUS proteinopathy. Protein Cell 2: 141–149. 21327870 10.1007/s13238-011-1014-5 PMC3093303

[48] Dormann D, Rodde R, Edbauer D, Bentmann E, Fischer I, et al (2010) ALS-associated fused in sarcoma (FUS) mutations disrupt Transportin-mediated nuclear import. EMBO J 29: 2841–2857. 20606625 10.1038/emboj.2010.143 PMC2924641

[49] Shelkovnikova TA, Ustyugov AA, Smirnov AP, Skvortsova VI, Buchman VL, et al (2011) FUS gene mutations associated with familiar forms of amyotrophic lateral sclerosis affect cellular localization and aggregation properties of the encoded protein. Dokl Biochem Biophys 438: 123–126. 21725888 10.1134/S1607672911030045

[50] Elbers CC, van Eijk KR, Franke L, Mulder F, van der Schouw YT, et al (2009) Using genome-wide pathway analysis to unravel the etiology of complex diseases. Genet Epidemiol 33: 419–431. 19235186 10.1002/gepi.20395

[51] Bosco DA, Lemay N, Ko HK, Zhou H, Burke C, et al (2010) Mutant FUS proteins that cause amyotrophic lateral sclerosis incorporate into stress granules. Hum Mol Genet 19: 4160–4175. 20699327 10.1093/hmg/ddq335 PMC2981014

