# Supplementary material for: Characterization of FUS Mutations in Amyotrophic Lateral Sclerosis Using RNA-Seq
Source: PLoS One. 2013 Apr 8;8(4):e60788. doi: 10.1371/journal.pone.0060788 (PMC3620060; doi:10.1371/journal.pone.0060788)

Supporting Information

Characterization of FUS mutations in amyotrophic lateral sclerosis using RNA-Seq

Marka van Blitterswijk, Eric T. Wang, Brad A. Friedman, Pamela J. Keagle, Patrick Lowe, Ashley Lyn Leclerc, Leonard H. van den Berg, David E. Housman, Jan H. Veldink, John E. Landers

Materials and Methods S1. Formulas used for RNA-Seq analysis.

Table S1. Summary of RNA-Seq analysis.

Table S2. Functional annotation KEGG pathways.

Table S3. Differentially expressed genes present within identified KEGG pathways.

Table S4. Significant protein domains present in differentially expressed genes.

Table S5. Shared genes with retained introns as identified by Venn diagrams and KEGG pathways.

Figure S1. MA plots per condition comparison.

Figure S2. *FUS* gene expression levels measured by RNA-Seq.

Figure S3. FUS protein levels measured by western blot.

Figure S4. Correlation of RNA-Seq and RT-PCR for *RBM25*, *TAF15*, *TARS*, *TPR*, *APP* and *HN1*.

Figure S5. Correlation of RNA-Seq and Semi-quantitative PCR for *PRPF8* and *RPS24*.

Materials and Methods S1. Formulas used for RNA-Seq analysis.

Exon Skipping: Log_2 (Inclusion: Skip + General Ratio) in the two groups, respectively. In formula $LISGR1 = \text{Log}_2\ ((jup1 + eal1 + jdn1) / (eup1 + jsk1 + edn1))$ and analogously for LISGR2.

Intron Retention: Log_2 (Intron: Flank Ratio). Log-Odds-Ratio (Flanking). In formula $LORF = LIFR2 - LIFR1$. The sign convention means that higher value indicates more intron retention (unspliced isoform) in the second group. This indicates the level of the intron relative to the flanking exons.

More detailed information regarding terms and definitions can be found on the ExpressionPlot website (http://www.expressionplot.com/wiki/index.php?title=Project_directory_structure) and corresponding manuscript [17].

Table S1. Summary of RNA-Seq analysis.

Sample	*No. Reads*	*No. Reads Passing QC*	*% Passing QC*	*Uniquely Mapping*	*% Uniquely mapping*	*No. Ribosomal*	*% Ribosomal*	*Splice Junctions*	*% Splice Junctions*
Wild-type	31,051,162	31,022,757	99.91	21,865,318	70.48	446,094	2.04	2,222,084	10.16
R521G	31,077,360	31,057,750	99.94	21,710,924	69.91	488,727	2.25	2,292,605	10.56
R522G	24,491,122	24,476,034	99.94	17,427,598	71.20	474,659	2.72	1,754,345	10.07
siRNA	28,196,024	28,179,703	99.94	20,640,291	73.25	291,210	1.41	2,242,343	10.86
Vector	27,295,832	27,273,639	99.92	19,416,054	71.19	421,688	2.17	1,983,350	10.22
**Average**	28,422,300	28,401,977	99.93	20,212,037	71.20	424,476	2.12	2,098,945	10.37

Table S2. Functional annotation KEGG pathways.

Analysis	*Group*	*KEGG pathway*	*Count*	*P-value*	*Benjamini*
Differential expression	**FUS-WT vs. vector**	Ribosome	14	5.6E-6	7.2E-4
	**siRNA vs. vector**	Spliceosome	34	7.1E-11	1.2E-8
		Ribosome	26	1.9E-9	1.6E-7
	**R521G vs. vector**	Spliceosome	18	4.4E-10	4.1E-8
	**R522G vs. vector**	Ribosome	15	3.1E-8	3.1E-6
		Spliceosome	15	3.3E-6	1.7E-4
		Mismatch repair	6	2.4E-4	8.2E-3
		DNA replication	7	2.7E-4	6.8E-3
		Cell cycle	11	1.3E-3	2.6E-2
Skipped exons	**FUS-WT vs. vector**	N/A	N/A	N/A	N/A
	**siRNA vs. vector**	Ribosome	18	8.6E-11	8.9E-9
		Spliceosome	17	2.0E-7	1.1E-5
	**R521G vs. vector**	Ribosome	6	1.6E-4	6.7E-3
	**R522G vs. vector**	Spliceosome	6	3.7E-4	1.4E-2
		Ribosome	5	9.0E-4	1.6E-2
Retained introns	**FUS-WT vs. vector**	Spliceosome	50	1.0E-16	1.9E-14
		Huntington's disease	54	3.9E-12	3.3E-10
		Proteasome	21	2.7E-8	1.5E-6
		Parkinson's disease	37	4.9E-8	2.1E-6
		Oxidative phosphorylation	37	7.6E-8	2.6E-6
		Cell cycle	36	8.6E-8	2.4E-6
		DNA replication	17	3.3E-7	7.9E-6
		Pyrimidine metabolism	29	5.5E-7	1.2E-5
		RNA polymerase	14	2.3E-6	4.4E-5
		Aminoacyl-tRNA biosynthesis	17	2.7E-6	4.6E-5
		Alzheimer's disease	38	9.9E-6	1.5E-4
		Nucleotide excision repair	16	3.8E-5	5.3E-4
		Citrate cycle (TCA cycle)	13	5.4E-5	7.0E-4
		Purine metabolism	33	2.1E-4	2.5E-3
		Mismatch repair	10	4.3E-4	4.9E-3
		RNA degradation	15	2.8E-3	2.9E-2
	**siRNA vs. vector**	Spliceosome	57	1.4E-22	2.4E-20
		Huntington's disease	54	2.4E-12	2.0E-10

		Pyrimidine metabolism	30	1.1E-7	6.4E-6
		Proteasome	20	1.3E-7	5.6E-6
		Cell cycle	35	2.1E-7	7.1E-6
		DNA replication	17	2.8E-7	7.9E-6
		Parkinson's disease	35	3.9E-7	9.5E-6
		Oxidative phosphorylation	35	5.8E-7	1.2E-5
		RNA polymerase	14	2.0E-6	3.9E-5
		Aminoacyl-tRNA biosynthesis	17	2.3E-6	4.0E-5
		Alzheimer's disease	37	1.9E-5	2.9E-4
		Citrate cycle (TCA cycle)	13	4.8E-5	6.8E-4
		Purine metabolism	34	6.9E-5	9.1E-4
		Mismatch repair	10	3.9E-4	4.8E-3
		Nucleotide excision repair	14	5.4E-4	6.2E-3
	**R521G vs. vector**	Spliceosome	48	4.7E-16	7.5E-14
		Huntington's disease	49	2.9E-10	2.5E-8
		Proteasome	21	1.1E-8	6.0E-7
		DNA replication	18	2.0E-8	8.7E-7
		Cell cycle	36	2.2E-8	7.4E-7
		Pyrimidine metabolism	28	6.7E-7	1.9E-5
		Oxidative phosphorylation	33	2.1E-6	5.2E-5
		Aminoacyl-tRNA biosynthesis	16	7.4E-6	1.6E-4
		RNA polymerase	13	9.2E-6	1.7E-4
		Parkinson's disease	31	1.3E-5	2.2E-4
		Alzheimer's disease	34	1.1E-4	1.8E-3
		Purine metabolism	32	1.8E-4	2.6E-3
		Mismatch repair	10	2.9E-4	3.8E-3
		Nucleotide excision repair	14	3.6E-4	4.4E-3
		RNA degradation	15	1.7E-3	1.9E-2
	**R522G vs. vector**	Spliceosome	46	4.3E-16	7.3E-14
		Huntington's disease	47	1.5E-10	1.2E-8
		Proteasome	20	1.5E-8	8.4E-7
		Pyrimidine metabolism	29	2.5E-8	1.0E-6
		Cell cycle	34	2.9E-8	9.4E-7
		DNA replication	16	3.3E-7	8.9E-6
		RNA polymerase	14	4.4E-7	1.0E-5
		Purine metabolism	33	1.2E-5	2.5E-4

		Aminoacyl-tRNA biosynthesis	15	1.3E-5	2.4E-4
		Parkinson's disease	29	1.8E-5	2.9E-4
		Oxidative phosphorylation	27	1.7E-4	2.6E-3
		Alzheimer's disease	30	6.1E-4	8.3E-3
		Citrate cycle (TCA cycle)	10	1.8E-3	2.2E-2
		Nucleotide excision repair	12	2.2E-3	2.5E-2
		RNA degradation	14	2.2E-3	2.4E-2

Abbreviations: FUS-WT = cell lines transfected with human FUS wild-type, used as a model for overexpression, siRNA = cell lines transfected with FUS small interfering RNA (siRNA), used as a model for underexpression, R521G = p.Arg521Gly, R522G = p.Arg522Gly, and N/A = not applicable. Significant KEGG pathways are shown for each condition. *Homo sapiens* is used as background, in Table 1 the Vector alone is used as background. Genes are specified in Table S3.

Table S3. Differentially expressed genes present within identified KEGG pathways.

Group	*KEGG pathway*	*Name*	*Description*
**FUS-WT vs. vector**	Ribosome	RPL13	ribosomal protein L13
		RPL18	ribosomal protein L18
		RPL26	ribosomal protein L26
		RPL29	ribosomal protein L29
		RPL37A	ribosomal protein L37a
		RPL8	ribosomal protein L8
		RPS11	ribosomal protein S11
		RPS16	ribosomal protein S16
		RPS19	ribosomal protein S19
		RPS3	ribosomal protein S3
		RPS4X	ribosomal protein S4X
		RPS6	ribosomal protein S6
		RPLP0	ribosomal protein, large, P0
		RPLP2	ribosomal protein, large, P2
**siRNA vs. vector**	Spliceosome	DDX23	DEAD (Asp-Glu-Ala-Asp) box polypeptide 23
		DDX46	DEAD (Asp-Glu-Ala-Asp) box polypeptide 46
		BAT1	HLA-B associated transcript 1
		LSM2	LSM2 homolog, U6 small nuclear RNA associated
		PRP19	PRP19/PSO4 pre-mRNA processing factor 19 homolog
		PRPF40A	PRP40 pre-mRNA processing factor 40 homolog A
		PRPF8	PRP8 pre-mRNA processing factor 8 homolog
		RBM17	RNA-binding motif protein 17
		RBM25	RNA-binding motif protein 25
		THOC2	THO complex 2
		THOC4	THO complex 4
		U2AFBP	U2 small nuclear RNA auxiliary factor 1
		SR140	U2-associated SR140 protein
		ACIN1	apoptotic chromatin condensation inducer 1
		EFTUD2	elongation factor Tu GTP binding domain containing 2
		HSPA1A	heat shock 70kDa protein 1B
		HSPA8	heat shock 70kDa protein 8

		HNRNPA1	heterogeneous nuclear ribonucleoprotein A1-like 3
		HNRNPM	heterogeneous nuclear ribonucleoprotein M
		HNRNPU	heterogeneous nuclear ribonucleoprotein U
		SNRNP200	similar to U5 snRNP-specific protein, 200 kDa
		SNRPD1	small nuclear ribonucleoprotein D1 polypeptide 16kDa
		SNRPB	small nuclear ribonucleoprotein polypeptides B and B1
		SF3B1	splicing factor 3b, subunit 1, 155kDa
		SF3B2	splicing factor 3b, subunit 2, 145kDa
		SFRS1	splicing factor, arginine/serine-rich 1
		SFRS2	splicing factor, arginine/serine-rich 2
		SFRS4	splicing factor, arginine/serine-rich 4
		SFRS6	splicing factor, arginine/serine-rich 6
		SFRS7	splicing factor, arginine/serine-rich 7, 35kDa
		TXNL4A	thioredoxin-like 4A
		TCERG1	transcription elongation regulator 1
		USP39	ubiquitin specific peptidase 39
		ZMAT2	zinc finger, matrin type 2
	Ribosome	RPL10	ribosomal protein L10
		RPL10A	ribosomal protein L10a
		RPL13	ribosomal protein L13
		RPL14	ribosomal protein L14
		RPL15	ribosomal protein L15
		RPL18	ribosomal protein L18
		RPL23	ribosomal protein L23
		RPL24	ribosomal protein L24
		RPL26	ribosomal protein L26
		RPL28	ribosomal protein L28
		RPL29	ribosomal protein L29
		RPL30	ribosomal protein L30
		RPL35A	ribosomal protein L35a
		RPL36AL	ribosomal protein L36a-like
		RPL38	ribosomal protein L38
		RPL3	ribosomal protein L3
		RPL5	ribosomal protein L5
		RPL7A	ribosomal protein L7a
		RPS11	ribosomal protein S11

		RPS16	ribosomal protein S16
		RPS19	ribosomal protein S19
		RPS20	ribosomal protein S20
		RPS23	ribosomal protein S23
		RPS29	ribosomal protein S29
		RPS3	ribosomal protein S3
		RPS6	ribosomal protein S6
**R521G vs. vector**	Spliceosome	DDX23	DEAD (Asp-Glu-Ala-Asp) box polypeptide 23
		PRPF8	PRP8 pre-mRNA processing factor 8 homolog
		RBM25	RNA-binding motif protein 25
		THOC4	THO complex 4
		U2AF2	U2 small nuclear RNA auxiliary factor 2
		SR140	U2-associated SR140 protein
		ACIN1	apoptotic chromatin condensation inducer 1
		CTBL1	catenin, beta like 1
		HSPA8	heat shock 70kDa protein 8
		HNRNPU	heterogeneous nuclear ribonucleoprotein U
		PCBP1	poly(rC) binding protein 1
		PRPF6	similar to U5 snRNP-associated 102 kDa protein
		SNRNP200	similar to U5 snRNP-specific protein, 200 kDa
		SF3A1	splicing factor 3a, subunit 1, 120kDa
		SFRS4	splicing factor, arginine/serine-rich 4
		SART1	squamous cell carcinoma antigen recognized by T cells
		TCERG1	transcription elongation regulator 1
		ZMAT2	zinc finger, matrin type 2
**R522G vs. vector**	Spliceosome	CDC5L	CDC5 cell division cycle 5-like
		DDX46	DEAD (Asp-Glu-Ala-Asp) box polypeptide 46
		DDX5	DEAD (Asp-Glu-Ala-Asp) box polypeptide 5
		DDX15	DEAH (Asp-Glu-Ala-His) box polypeptide 15
		FUSIP1	FUS interacting protein (serine/arginine-rich) 1
		PRPF38B	PRP38 pre-mRNA processing factor 38 (yeast) domain containing B
		THOC2	THO complex 2
		HNRNPM	heterogeneous nuclear ribonucleoprotein M
		HNRNPU	heterogeneous nuclear ribonucleoprotein U
		NCBP1	nuclear cap binding protein subunit 1, 80kDa
		RNRPB	small nuclear ribonucleoprotein polypeptides B and B1

		SF3B1	splicing factor 3b, subunit 1, 155kDa
		SFRS2	splicing factor, arginine/serine-rich 2
		SFRS5	splicing factor, arginine/serine-rich 5
		SFRS7	splicing factor, arginine/serine-rich 7, 35kDa
	Ribosome	RPL10	ribosomal protein L10
		RPL11	ribosomal protein L11
		RPL23	ribosomal protein L23
		RPL26	ribosomal protein L26
		RPL29	ribosomal protein L29
		RPL34	ribosomal protein L34
		RPL4	ribosomal protein L4
		RPS16	ribosomal protein S16
		RPS19	ribosomal protein S19
		RPS20	ribosomal protein S20
		RPS24	ribosomal protein S24
		RPS29	ribosomal protein S29
		RPS3	ribosomal protein S3
		RPS4X	ribosomal protein S4, X-linked
		RPS6	ribosomal protein S6
	Mismatch repair	EXO1	exonuclease 1
		MSH2	mutS homolog 2, colon cancer, nonpolyposis type 1
		PCNA	proliferating cell nuclear antigen
		RFC1	replication factor C (activator 1) 1, 145kDa
		RFC5	replication factor C (activator 1) 5, 36.5kDa
		SSBP1	single-stranded DNA-binding protein 1
	DNA replication	MCM4	minichromosome maintenance complex component 4
		MCM6	minichromosome maintenance complex component 6
		MCM7	minichromosome maintenance complex component 7
		PCNA	proliferating cell nuclear antigen
		RFC1	replication factor C (activator 1) 1, 145kDa
		RFC5	replication factor C (activator 1) 5, 36.5kDa
		SSBP1	single-stranded DNA-binding protein 1
	Cell cycle	CDC25A	cell division cycle 25 homolog A (S. pombe)
		HDAC2	histone deacetylase 2
		MCM4	minichromosome maintenance complex component 4
		MCM6	minichromosome maintenance complex component 6

		MCM7	minichromosome maintenance complex component 7
		PLK1	polo-like kinase 1 (Drosophila)
		PCNA	proliferating cell nuclear antigen
		YWHAE	similar to 14-3-3 protein epsilon (14-3-3E)
		PRKDC	similar to protein kinase, DNA-activated, catalytic polypeptide
		STAG2	stromal antigen 2
		YWHAQ	tyrosine 3-monooxygenase/tryptophan 5-monooxygenase activation protein, theta polypeptide

Table S4. Significant protein domains present in differentially expressed genes.

Group	*INTERPRO*	*Count*	*P-value*	*Benjamini*
**FUS-WT vs. vector**	RNA recognition motif, RNP-1	20	7.2E-7	6.0E-4
	Endoplasmic reticulum, targeting sequence	10	9.2E-7	3.8E-4
	Nucleotide-binding, alpha-beta plait	17	5.2E-5	1.4E-2
**siRNA vs. vector**	Nucleotide-binding, alpha-beta plait	48	3.2E-14	5.4E-11
	RNA recognition motif, RNP-1	46	4.1E-13	3.5E-10
	Zinc finger, PHD-type	19	1.2E-5	6.6E-3
	Zinc finger, PHD-finger	18	2.1E-5	8.6E-3
	Helicase, superfamily 1 and 2, ATP-binding	20	4.0E-5	1.3E-2
	DEAD-like helicase, N-terminal	20	5.2E-5	1.5E-2
	Actinin-type, actin-binding, conserved site	9	5.4E-5	1.3E-2
	Zinc finger, PHD-type, conserved site	18	6.9E-5	1.4E-2
	K Homology	11	7.4E-5	1.4E-2
	DNA/RNA helicase, C-terminal	19	1.3E-4	2.2E-2
**R521G vs. vector**	RNA recognition motif, RNP-1	28	1.9E-15	1.2E-12
	Nucleotide-binding, alpha-beta plait	27	2.0E-14	6.3E-12
	DNA/RNA helicase, C-terminal	12	4.3E-6	8.9E-4
	Helicase, superfamily 1 and 2, ATP-binding	12	4.3E-6	8.9E-4
	DEAD-like helicase, N-terminal	12	5.2E-6	8.0E-4
	Zinc finger, PHD-finger	10	2.6E-5	3.2E-3
	Zinc finger, PHD-type	10	4.1E-5	4.2E-3
	DNA-binding SAP	6	5.2E-5	4.6E-3
	SNF2-related	6	2.7E-4	2.1E-2
	K Homology, type 1	6	3.1E-4	2.1E-2
	K Homology, type 1, subgroup	6	4.2E-4	2.5E-2
	K Homology	6	5.4E-4	3.0E-2
**R522G vs. vector**	Nucleotide-binding, alpha-beta plait	20	9.2E-9	6.3E-6
	RNA recognition motif, RNP-1	19	4.6E-8	1.6E-5
	HEAT	10	5.0E-7	1.1E-4
	Armadillo-like helical	13	3.1E-6	5.3E-4
	RNA helicase, ATP-dependent, DEAD-box, conserved site	7	1.9E-5	2.6E-3
	Chaperonin TCP-1, conserved site	5	1.9E-5	2.2E-3

	Helicase, superfamily 1 and 2, ATP-binding	11	2.1E-5	2.1E-3
	DNA/RNA helicase, C-terminal	11	2.1E-5	2.1E-3
	DEAD-like helicase, N-terminal	11	2.5E-5	2.1E-3
	Chaperone, tailless complex polypeptide 1	5	3.0E-5	2.2E-3
	Nucleic acid-binding, OB-fold	8	4.1E-5	2.8E-3
	RNA helicase, DEAD-box type, Q motif	7	4.5E-5	2.8E-3
	DNA/RNA helicase, DEAD/DEAH box type, N-terminal	8	1.0E-4	5.8E-3
	Importin-beta, N-terminal	5	1.2E-4	6.1E-3
	Chaperonin Cpn60/TCP-1	5	1.2E-4	6.1E-3
	DNA-dependent ATPase MCM, conserved site	4	2.9E-4	1.4E-2
	DNA-dependent ATPase MCM	4	4.4E-4	2.0E-2
	ATPase, F1/V1/A1 complex, alpha/beta subunit, nucleotide-binding domain, active site	4	1.1E-3	4.6E-2
	Heat shock protein Hsp90	4	1.1E-3	4.6E-2

Table S5. Shared genes with retained introns as identified by Venn diagrams and KEGG pathways.

KEGG pathway	Name	Description
Spliceosome	BUD31	BUD31 homolog (S. cerevisiae)
	DDX23	DEAD (Asp-Glu-Ala-Asp) box polypeptide 23
	DDX5	DEAD (Asp-Glu-Ala-Asp) box polypeptide 5
	DHX38	DEAH (Asp-Glu-Ala-His) box polypeptide 38
	LSM4	LSM4 homolog, U6 small nuclear RNA associated (S. cerevisiae)
	LSM7	LSM7 homolog, U6 small nuclear RNA associated (S. cerevisiae)
	PRPF3	PRP3 pre-mRNA processing factor 3 homolog (S. cerevisiae)
	PRPF31	PRP31 pre-mRNA processing factor 31 homolog (S. cerevisiae)
	PRPF38B	PRP38 pre-mRNA processing factor 38 (yeast) domain containing B
	PRPF8	PRP8 pre-mRNA processing factor 8 homolog (S. cerevisiae)
	RBM17	RNA binding motif protein 17
	RBM25	RNA binding motif protein 25
	THOC4	THO complex 4
	U2AF1	U2 small nuclear RNA auxiliary factor 1
	U2AF2	U2 small nuclear RNA auxiliary factor 2
	ACIN1	apoptotic chromatin condensation inducer 1
	CTNNBL1	catenin, beta like 1
	EFTUD2	elongation factor Tu GTP binding domain containing 2
	EIF4A3	eukaryotic translation initiation factor 4A, isoform 3
	HSPA8	heat shock 70kDa protein 8
	HNRPM	heterogeneous nuclear ribonucleoprotein M
	SNRPG	hypothetical protein LOC100132425; similar to small nuclear ribonucleoprotein polypeptide G; small nuclear ribonucleoprotein polypeptide G; small nuclear ribonucleoprotein G-like protein
	PQBP1	polyglutamine binding protein 1
	PRPF6	similar to U5 snRNP-associated 102 kDa protein (U5-102 kDa protein); PRP6 pre-mRNA processing factor 6 homolog (S. cerevisiae)
	ASCC3L1	similar to U5 snRNP-specific protein, 200 kDa; small nuclear ribonucleoprotein 200kDa (U5)

	WDR57	small nuclear ribonucleoprotein 40kDa (U5)
	SNRPA	small nuclear ribonucleoprotein polypeptide A
	SF3B14	splicing factor 3B, 14 kDa subunit
	SF3B1	splicing factor 3b, subunit 1, 155kDa
	SF3B2	splicing factor 3b, subunit 2, 145kDa
	SF3B3	splicing factor 3b, subunit 3, 130kDa
	SFRS1	splicing factor, arginine/serine-rich 1
	SFRS2	splicing factor, arginine/serine-rich 2
	SFRS4	splicing factor, arginine/serine-rich 4
	SFRS5	splicing factor, arginine/serine-rich 5
	SFRS7	splicing factor, arginine/serine-rich 7, 35kDa
	SFRS9	splicing factor, arginine/serine-rich 9
	SART1	squamous cell carcinoma antigen recognized by T cells
	SMNDC1	survival motor neuron domain containing 1
	TCERG1	transcription elongation regulator 1
	TRA2A	transformer 2 alpha homolog (Drosophila)
	SFRS10	transformer 2 beta homolog (Drosophila)
	USP39	ubiquitin specific peptidase 39
Huntington's disease	ATP5G3	ATP synthase, H+ transporting, mitochondrial F0 complex, subunit C3 (subunit 9)
	BAX	BCL2-associated X protein
	CREBBP	CREB binding protein
	NDUFA1	NADH dehydrogenase (ubiquinone) 1 alpha subcomplex, 1, 7.5kDa
	NDUFA10	NADH dehydrogenase (ubiquinone) 1 alpha subcomplex, 10, 42kDa
	NDUFA2	NADH dehydrogenase (ubiquinone) 1 alpha subcomplex, 2, 8kDa
	NDUFA8	NADH dehydrogenase (ubiquinone) 1 alpha subcomplex, 8, 19kDa
	NDUFB10	NADH dehydrogenase (ubiquinone) 1 beta subcomplex, 10, 22kDa
	NDUFS1	NADH dehydrogenase (ubiquinone) Fe-S protein 1, 75kDa (NADH-coenzyme Q reductase)
	NDUFS3	NADH dehydrogenase (ubiquinone) Fe-S protein 3, 30kDa (NADH-coenzyme Q reductase)
	NDUFS6	NADH dehydrogenase (ubiquinone) Fe-S protein 6, 13kDa (NADH-coenzyme Q reductase)
	NDUFS7	NADH dehydrogenase (ubiquinone) Fe-S protein 7, 20kDa (NADH-coenzyme Q reductase)

	NDUFV1	NADH dehydrogenase (ubiquinone) flavoprotein 1, 51kDa
	AP2A1	adaptor-related protein complex 2, alpha 1 subunit
	AP2A2	adaptor-related protein complex 2, alpha 2 subunit
	AP2M1	adaptor-related protein complex 2, mu 1 subunit
	CLTC	clathrin, heavy chain (Hc)
	CLTA	clathrin, light chain (Lca)
	CLTB	clathrin, light chain (Lcb)
	COX4I1	cytochrome c oxidase subunit IV isoform 1
	COX7B	cytochrome c oxidase subunit VIIb
	COX6B1	cytochrome c oxidase subunit Vib polypeptide 1 (ubiquitous)
	CYC1	cytochrome c-1
	HDAC1	histone deacetylase 1
	HDAC2	histone deacetylase 2
	IFT57	intraflagellar transport 57 homolog (Chlamydomonas)
	POLR2A	polymerase (RNA) II (DNA directed) polypeptide A, 220kDa
	POLR2B	polymerase (RNA) II (DNA directed) polypeptide B, 140kDa
	POLR2E	polymerase (RNA) II (DNA directed) polypeptide E, 25kDa
	POLR2F	polymerase (RNA) II (DNA directed) polypeptide F
	POLR2G	polymerase (RNA) II (DNA directed) polypeptide G
	POLR2H	polymerase (RNA) II (DNA directed) polypeptide H
	POLR2J	polymerase (RNA) II (DNA directed) polypeptide J, 13.3kDa
	NDUFS5	similar to NADH dehydrogenase (ubiquinone) Fe-S protein 5, 15kDa (NADH-coenzyme Q reductase); NADH dehydrogenase (ubiquinone) Fe-S protein 5, 15kDa (NADH-coenzyme Q reductase)
	SLC25A5	solute carrier family 25 (mitochondrial carrier; adenine nucleotide translocator), member 5; solute carrier family 25 (mitochondrial carrier; adenine nucleotide translocator), member 5 pseudogene 8
	SDHA	succinate dehydrogenase complex, subunit A, flavoprotein (Fp)
	SDHB	succinate dehydrogenase complex, subunit B, iron sulfur (Ip)
	SOD1	superoxide dismutase 1, soluble
	UCRC	ubiquinol-cytochrome c reductase complex (7.2 kD)
	UQCRC1	ubiquinol-cytochrome c reductase core protein I
	UQCRC2	ubiquinol-cytochrome c reductase core protein II
	VDAC3	voltage-dependent anion channel 3
DNA replication	MCM2	minichromosome maintenance complex component 2
	MCM3	minichromosome maintenance complex component 3
	MCM4	minichromosome maintenance complex component 4

	MCM5	minichromosome maintenance complex component 5
	MCM6	minichromosome maintenance complex component 6
	MCM7	minichromosome maintenance complex component 7
	POLD1	polymerase (DNA directed), delta 1, catalytic subunit 125kDa
	POLD2	polymerase (DNA directed), delta 2, regulatory subunit 50kDa
	POLE	polymerase (DNA directed), epsilon
	POLE3	polymerase (DNA directed), epsilon 3 (p17 subunit)
	PCNA	proliferating cell nuclear antigen
	RFC2	replication factor C (activator 1) 2, 40kDa
	RFC5	replication factor C (activator 1) 5, 36.5kDa
	RPA1	replication protein A1, 70kDa
	RPA2	replication protein A2, 32kDa
	RNASEH2A	ribonuclease H2, subunit A
Proteasome	PSMC3	proteasome (prosome, macropain) 26S subunit, ATPase, 3
	PSMD1	proteasome (prosome, macropain) 26S subunit, non-ATPase, 1
	PSMD11	proteasome (prosome, macropain) 26S subunit, non-ATPase, 11
	PSMD13	proteasome (prosome, macropain) 26S subunit, non-ATPase, 13
	PSMD14	proteasome (prosome, macropain) 26S subunit, non-ATPase, 14
	PSMD3	proteasome (prosome, macropain) 26S subunit, non-ATPase, 3
	PSMD7	proteasome (prosome, macropain) 26S subunit, non-ATPase, 7
	PSMD8	proteasome (prosome, macropain) 26S subunit, non-ATPase, 8
	PSME3	proteasome (prosome, macropain) activator subunit 3 (PA28 gamma; Ki)
	PSMA1	proteasome (prosome, macropain) subunit, alpha type, 1
	PSMA5	proteasome (prosome, macropain) subunit, alpha type, 5
	PSMA7	proteasome (prosome, macropain) subunit, alpha type, 7
	PSMB1	proteasome (prosome, macropain) subunit, beta type, 1
	PSMB2	proteasome (prosome, macropain) subunit, beta type, 2
	PSMB4	proteasome (prosome, macropain) subunit, beta type, 4
	PSMB6	proteasome (prosome, macropain) subunit, beta type, 6
	PSMB7	proteasome (prosome, macropain) subunit, beta type, 7
	PSMC4	similar to 26S protease regulatory subunit 6B (MIP224) (MB67-interacting protein) (TAT-binding protein 7) (TBP-7); proteasome (prosome, macropain) 26S subunit, ATPase, 4
Pyrimidine metabolism	ITPA	inosine triphosphatase (nucleoside triphosphate pyrophosphatase)

	NME1-NME2	non-metastatic cells 1, protein (NM23A) expressed in; NME1-NME2 readthrough transcript; non-metastatic cells 2, protein (NM23B) expressed in
	NME3	non-metastatic cells 3, protein expressed in
	NP	nucleoside phosphorylase
	POLD1	polymerase (DNA directed), delta 1, catalytic subunit 125kDa
	POLD2	polymerase (DNA directed), delta 2, regulatory subunit 50kDa
	POLE	polymerase (DNA directed), epsilon
	POLE3	polymerase (DNA directed), epsilon 3 (p17 subunit)
	POLR1A	polymerase (RNA) I polypeptide A, 194kDa
	POLR1C	polymerase (RNA) I polypeptide C, 30kDa
	POLR2A	polymerase (RNA) II (DNA directed) polypeptide A, 220kDa
	POLR2B	polymerase (RNA) II (DNA directed) polypeptide B, 140kDa
	POLR2E	polymerase (RNA) II (DNA directed) polypeptide E, 25kDa
	POLR2F	polymerase (RNA) II (DNA directed) polypeptide F
	POLR2G	polymerase (RNA) II (DNA directed) polypeptide G
	POLR2H	polymerase (RNA) II (DNA directed) polypeptide H
	POLR2J	polymerase (RNA) II (DNA directed) polypeptide J, 13.3kDa
	POLR3A	polymerase (RNA) III (DNA directed) polypeptide A, 155kDa
	POLR3H	polymerase (RNA) III (DNA directed) polypeptide H (22.9kD)
	POLR3K	polymerase (RNA) III (DNA directed) polypeptide K, 12.3 kDa
	TXNRD2	thioredoxin reductase 2
	TK1	thymidine kinase 1, soluble
	TYMS	thymidylate synthetase
	UMPS	uridine monophosphate synthetase
	UCK2	uridine-cytidine kinase 2
RNA polymerase	POLR1A	polymerase (RNA) I polypeptide A, 194kDa
	POLR1C	polymerase (RNA) I polypeptide C, 30kDa
	POLR2A	polymerase (RNA) II (DNA directed) polypeptide A, 220kDa
	POLR2B	polymerase (RNA) II (DNA directed) polypeptide B, 140kDa
	POLR2E	polymerase (RNA) II (DNA directed) polypeptide E, 25kDa
	POLR2F	polymerase (RNA) II (DNA directed) polypeptide F
	POLR2G	polymerase (RNA) II (DNA directed) polypeptide G
	POLR2H	polymerase (RNA) II (DNA directed) polypeptide H
	POLR2J	polymerase (RNA) II (DNA directed) polypeptide J, 13.3kDa

	POLR3A	polymerase (RNA) III (DNA directed) polypeptide A, 155kDa
	POLR3H	polymerase (RNA) III (DNA directed) polypeptide H (22.9kD)
	POLR3K	polymerase (RNA) III (DNA directed) polypeptide K, 12.3 kDa
Cell cycle	CDC45L	CDC45 cell division cycle 45-like (S. cerevisiae)
	CREBBP	CREB binding protein
	E2F1	E2F transcription factor 1
	RAD21	RAD21 homolog (S. pombe)
	ANAPC5	anaphase promoting complex subunit 5
	BUB3	budding uninhibited by benzimidazoles 3 homolog (yeast)
	CDC25A	cell division cycle 25 homolog A (S. pombe)
	CCNA2	cyclin A2
	CCNB1	cyclin B1
	CCNB2	cyclin B2
	CDK4	cyclin-dependent kinase 4
	CDKN2A	cyclin-dependent kinase inhibitor 2A (melanoma, p16, inhibits CDK4)
	HDAC1	histone deacetylase 1
	HDAC2	histone deacetylase 2
	MCM2	minichromosome maintenance complex component 2
	MCM3	minichromosome maintenance complex component 3
	MCM4	minichromosome maintenance complex component 4
	MCM5	minichromosome maintenance complex component 5
	MCM6	minichromosome maintenance complex component 6
	MCM7	minichromosome maintenance complex component 7
	PLK1	polo-like kinase 1 (Drosophila)
	PCNA	proliferating cell nuclear antigen
	RBX1	ring-box 1
	SMC1A	structural maintenance of chromosomes 1A
	TGFB1	transforming growth factor, beta 1
Parkinson's disease	ATP5G3	ATP synthase, H+ transporting, mitochondrial F0 complex, subunit C3 (subunit 9)
	NDUFA1	NADH dehydrogenase (ubiquinone) 1 alpha subcomplex, 1, 7.5kDa
	NDUFA10	NADH dehydrogenase (ubiquinone) 1 alpha subcomplex, 10, 42kDa
	NDUFA2	NADH dehydrogenase (ubiquinone) 1 alpha subcomplex, 2, 8kDa

	NDUFA8	NADH dehydrogenase (ubiquinone) 1 alpha subcomplex, 8, 19kDa
	NDUFB10	NADH dehydrogenase (ubiquinone) 1 beta subcomplex, 10, 22kDa
	NDUFS1	NADH dehydrogenase (ubiquinone) Fe-S protein 1, 75kDa (NADH-coenzyme Q reductase)
	NDUFS3	NADH dehydrogenase (ubiquinone) Fe-S protein 3, 30kDa (NADH-coenzyme Q reductase)
	NDUFS6	NADH dehydrogenase (ubiquinone) Fe-S protein 6, 13kDa (NADH-coenzyme Q reductase)
	NDUFS7	NADH dehydrogenase (ubiquinone) Fe-S protein 7, 20kDa (NADH-coenzyme Q reductase)
	NDUFV1	NADH dehydrogenase (ubiquinone) flavoprotein 1, 51kDa
	PARK7	Parkinson disease (autosomal recessive, early onset) 7
	COX4I1	cytochrome c oxidase subunit IV isoform 1
	COX7B	cytochrome c oxidase subunit VIIb
	COX6B1	cytochrome c oxidase subunit Vib polypeptide 1 (ubiquitous)
	CYC1	cytochrome c-1
	NDUFS5	similar to NADH dehydrogenase (ubiquinone) Fe-S protein 5, 15kDa (NADH-coenzyme Q reductase); NADH dehydrogenase (ubiquinone) Fe-S protein 5, 15kDa (NADH-coenzyme Q reductase)
	SLC25A5	solute carrier family 25 (mitochondrial carrier; adenine nucleotide translocator), member 5; solute carrier family 25 (mitochondrial carrier; adenine nucleotide translocator), member 5 pseudogene 8
	SDHA	succinate dehydrogenase complex, subunit A, flavoprotein (Fp)
	SDHB	succinate dehydrogenase complex, subunit B, iron sulfur (Ip)
	UCRC	ubiquinol-cytochrome c reductase complex (7.2 kD)
	UQCRC1	ubiquinol-cytochrome c reductase core protein I
	UQCRC2	ubiquinol-cytochrome c reductase core protein II
	UCHL1	ubiquitin carboxyl-terminal esterase L1 (ubiquitin thiolesterase)
	UBE2G1	ubiquitin-conjugating enzyme E2G 1 (UBC7 homolog, yeast)
	UBE2G2	ubiquitin-conjugating enzyme E2G 2 (UBC7 homolog, yeast)
	VDAC3	voltage-dependent anion channel 3
Purine metabolism	ATIC	5-aminoimidazole-4-carboxamide ribonucleotide formyltransferase/IMP cyclohydrolase
	IMPDH2	IMP (inosine monophosphate) dehydrogenase 2
	APRT	adenine phosphoribosyltransferase
	AK2	adenylate kinase 2
	HPRT1	hypoxanthine phosphoribosyltransferase 1
	ITPA	inosine triphosphatase (nucleoside triphosphate pyrophosphatase)

	NME1-NME2	non-metastatic cells 1, protein (NM23A) expressed in; NME1-NME2 readthrough transcript; non-metastatic cells 2, protein (NM23B) expressed in
	NME3	non-metastatic cells 3, protein expressed in
	NP	nucleoside phosphorylase
	NUDT5	nudix (nucleoside diphosphate linked moiety X)-type motif 5
	PRPS1	phosphoribosyl pyrophosphate synthetase 1; phosphoribosyl pyrophosphate synthetase 1-like 1
	PAICS	phosphoribosylaminoimidazole carboxylase, phosphoribosylaminoimidazole succinocarboxamide synthetase
	PFAS	phosphoribosylformylglycinamidine synthase
	POLD1	polymerase (DNA directed), delta 1, catalytic subunit 125kDa
	POLD2	polymerase (DNA directed), delta 2, regulatory subunit 50kDa
	POLE	polymerase (DNA directed), epsilon
	POLE3	polymerase (DNA directed), epsilon 3 (p17 subunit)
	POLR1A	polymerase (RNA) I polypeptide A, 194kDa
	POLR1C	polymerase (RNA) I polypeptide C, 30kDa
	POLR2A	polymerase (RNA) II (DNA directed) polypeptide A, 220kDa
	POLR2B	polymerase (RNA) II (DNA directed) polypeptide B, 140kDa
	POLR2E	polymerase (RNA) II (DNA directed) polypeptide E, 25kDa
	POLR2F	polymerase (RNA) II (DNA directed) polypeptide F
	POLR2G	polymerase (RNA) II (DNA directed) polypeptide G
	POLR2H	polymerase (RNA) II (DNA directed) polypeptide H
	POLR2J	polymerase (RNA) II (DNA directed) polypeptide J, 13.3kDa
	POLR3A	polymerase (RNA) III (DNA directed) polypeptide A, 155kDa
	POLR3H	polymerase (RNA) III (DNA directed) polypeptide H (22.9kD)
	POLR3K	polymerase (RNA) III (DNA directed) polypeptide K, 12.3 kDa
	PKM2	similar to Pyruvate kinase, isozymes M1/M2 (Pyruvate kinase muscle isozyme) (Cytosolic thyroid hormone-binding protein) (CTHBP) (THBP1); pyruvate kinase, muscle
Aminoacyl-tRNA biosynthesis	AARS	alanyl-tRNA synthetase
	NARS	asparaginyl-tRNA synthetase
	DARS	aspartyl-tRNA synthetase
	CARS	cysteinyl-tRNA synthetase
	QARS	glutaminyl-tRNA synthetase
	GARS	glycyl-tRNA synthetase
	LARS	leucyl-tRNA synthetase

	LARS2	leucyl-tRNA synthetase 2, mitochondrial
	MARS	methionyl-tRNA synthetase
	FARSA	phenylalanyl-tRNA synthetase, alpha subunit
	FARSB	phenylalanyl-tRNA synthetase, beta subunit
	SARS	seryl-tRNA synthetase
	TARS	threonyl-tRNA synthetase
	YARS	tyrosyl-tRNA synthetase
Oxidative phosphorylation	ATP5G3	ATP synthase, H+ transporting, mitochondrial F0 complex, subunit C3 (subunit 9)
	ATP5I	ATP synthase, H+ transporting, mitochondrial F0 complex, subunit E
	ATP6V1G1	ATPase, H+ transporting, lysosomal 13kDa, V1 subunit G1
	ATP6V1F	ATPase, H+ transporting, lysosomal 14kDa, V1 subunit F
	ATP6V1E1	ATPase, H+ transporting, lysosomal 31kDa, V1 subunit E1
	NDUFA1	NADH dehydrogenase (ubiquinone) 1 alpha subcomplex, 1, 7.5kDa
	NDUFA10	NADH dehydrogenase (ubiquinone) 1 alpha subcomplex, 10, 42kDa
	NDUFA2	NADH dehydrogenase (ubiquinone) 1 alpha subcomplex, 2, 8kDa
	NDUFA8	NADH dehydrogenase (ubiquinone) 1 alpha subcomplex, 8, 19kDa
	NDUFB10	NADH dehydrogenase (ubiquinone) 1 beta subcomplex, 10, 22kDa
	NDUFS1	NADH dehydrogenase (ubiquinone) Fe-S protein 1, 75kDa (NADH-coenzyme Q reductase)
	NDUFS3	NADH dehydrogenase (ubiquinone) Fe-S protein 3, 30kDa (NADH-coenzyme Q reductase)
	NDUFS6	NADH dehydrogenase (ubiquinone) Fe-S protein 6, 13kDa (NADH-coenzyme Q reductase)
	NDUFS7	NADH dehydrogenase (ubiquinone) Fe-S protein 7, 20kDa (NADH-coenzyme Q reductase)
	NDUFV1	NADH dehydrogenase (ubiquinone) flavoprotein 1, 51kDa
	COX4I1	cytochrome c oxidase subunit IV isoform 1
	COX7B	cytochrome c oxidase subunit VIIb
	COX6B1	cytochrome c oxidase subunit Vib polypeptide 1 (ubiquitous)
	CYC1	cytochrome c-1
	NDUFS5	similar to NADH dehydrogenase (ubiquinone) Fe-S protein 5, 15kDa (NADH-coenzyme Q reductase); NADH dehydrogenase (ubiquinone) Fe-S protein 5, 15kDa (NADH-coenzyme Q reductase)
	SDHA	succinate dehydrogenase complex, subunit A, flavoprotein (Fp)
	SDHB	succinate dehydrogenase complex, subunit B, iron sulfur (Ip)
	UCRC	ubiquinol-cytochrome c reductase complex (7.2 kD)

	UQCRC1	ubiquinol-cytochrome c reductase core protein I
	UQCRC2	ubiquinol-cytochrome c reductase core protein II
Alzheimer's disease	ATP5G3	ATP synthase, H+ transporting, mitochondrial F0 complex, subunit C3 (subunit 9)
	NDUFA1	NADH dehydrogenase (ubiquinone) 1 alpha subcomplex, 1, 7.5kDa
	NDUFA10	NADH dehydrogenase (ubiquinone) 1 alpha subcomplex, 10, 42kDa
	NDUFA2	NADH dehydrogenase (ubiquinone) 1 alpha subcomplex, 2, 8kDa
	NDUFA8	NADH dehydrogenase (ubiquinone) 1 alpha subcomplex, 8, 19kDa
	NDUFB10	NADH dehydrogenase (ubiquinone) 1 beta subcomplex, 10, 22kDa
	NDUFS1	NADH dehydrogenase (ubiquinone) Fe-S protein 1, 75kDa (NADH-coenzyme Q reductase)
	NDUFS3	NADH dehydrogenase (ubiquinone) Fe-S protein 3, 30kDa (NADH-coenzyme Q reductase)
	NDUFS6	NADH dehydrogenase (ubiquinone) Fe-S protein 6, 13kDa (NADH-coenzyme Q reductase)
	NDUFS7	NADH dehydrogenase (ubiquinone) Fe-S protein 7, 20kDa (NADH-coenzyme Q reductase)
	NDUFV1	NADH dehydrogenase (ubiquinone) flavoprotein 1, 51kDa
	APPBP1	NEDD8 activating enzyme E1 subunit 1
	APP	amyloid beta (A4) precursor protein
	APH1A	anterior pharynx defective 1 homolog A (C. elegans)
	CALM3	calmodulin 3 (phosphorylase kinase, delta); calmodulin 2 (phosphorylase kinase, delta); calmodulin 1 (phosphorylase kinase, delta)
	CAPN2	calpain 2, (m/II) large subunit
	COX4I1	cytochrome c oxidase subunit IV isoform 1
	COX7B	cytochrome c oxidase subunit VIIb
	COX6B1	cytochrome c oxidase subunit Vib polypeptide 1 (ubiquitous)
	CYC1	cytochrome c-1
	HSD17B10	hydroxysteroid (17-beta) dehydrogenase 10
	MAPK1	mitogen-activated protein kinase 1
	NDUFS5	similar to NADH dehydrogenase (ubiquinone) Fe-S protein 5, 15kDa (NADH-coenzyme Q reductase); NADH dehydrogenase (ubiquinone) Fe-S protein 5, 15kDa (NADH-coenzyme Q reductase)
	SDHA	succinate dehydrogenase complex, subunit A, flavoprotein (Fp)
	SDHB	succinate dehydrogenase complex, subunit B, iron sulfur (Ip)
	UCRC	ubiquinol-cytochrome c reductase complex (7.2 kD)
	UQCRC1	ubiquinol-cytochrome c reductase core protein I

	UQCRC2	ubiquinol-cytochrome c reductase core protein II
Nucleotide excision repair	DDB1	damage-specific DNA binding protein 1, 127kDa
	POLD1	polymerase (DNA directed), delta 1, catalytic subunit 125kDa
	POLD2	polymerase (DNA directed), delta 2, regulatory subunit 50kDa
	POLE	polymerase (DNA directed), epsilon
	POLE3	polymerase (DNA directed), epsilon 3 (p17 subunit)
	PCNA	proliferating cell nuclear antigen
	RFC2	replication factor C (activator 1) 2, 40kDa
	RFC5	replication factor C (activator 1) 5, 36.5kDa
	RPA1	replication protein A1, 70kDa
	RPA2	replication protein A2, 32kDa
	RBX1	ring-box 1
	XPC	xeroderma pigmentosum, complementation group C
Mismatch repair	MSH6	mutS homolog 6 (E. coli)
	POLD1	polymerase (DNA directed), delta 1, catalytic subunit 125kDa
	POLD2	polymerase (DNA directed), delta 2, regulatory subunit 50kDa
	PCNA	proliferating cell nuclear antigen
	RFC2	replication factor C (activator 1) 2, 40kDa
	RFC5	replication factor C (activator 1) 5, 36.5kDa
	RPA1	replication protein A1, 70kDa
	RPA2	replication protein A2, 32kDa
Citrate cycle (TCA cycle)	ACLY	ATP citrate lyase
	IDH3A	isocitrate dehydrogenase 3 (NAD+) alpha
	IDH3B	isocitrate dehydrogenase 3 (NAD+) beta
	IDH3G	isocitrate dehydrogenase 3 (NAD+) gamma
	MDH2	malate dehydrogenase 2, NAD (mitochondrial)
	PDHA1	pyruvate dehydrogenase (lipoamide) alpha 1
	SDHA	succinate dehydrogenase complex, subunit A, flavoprotein (Fp)
	SDHB	succinate dehydrogenase complex, subunit B, iron sulfur (Ip)
	SUCLG1	succinate-CoA ligase, alpha subunit

Figure S1. MA plots per condition comparison.

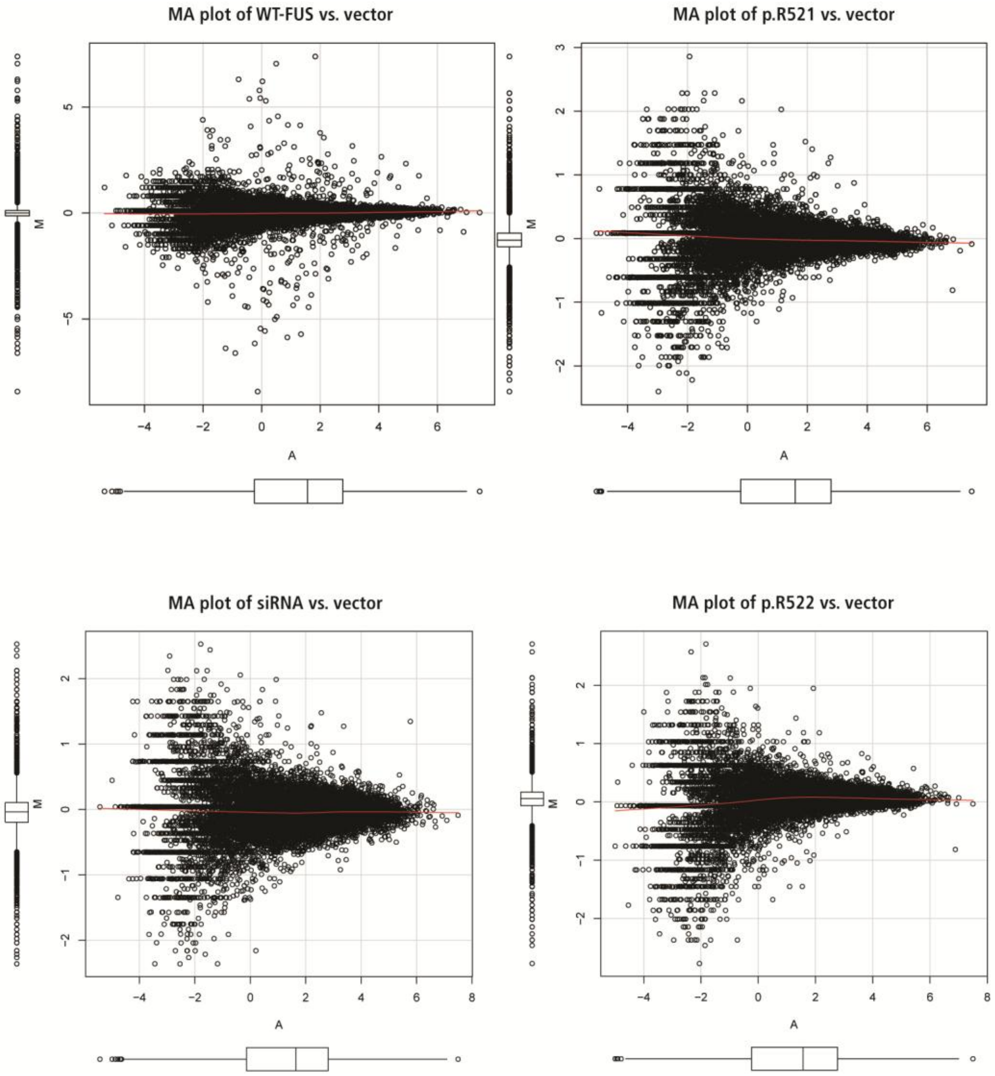

Figure S2. ***FUS*** **gene expression levels measured by RNA-Seq**.

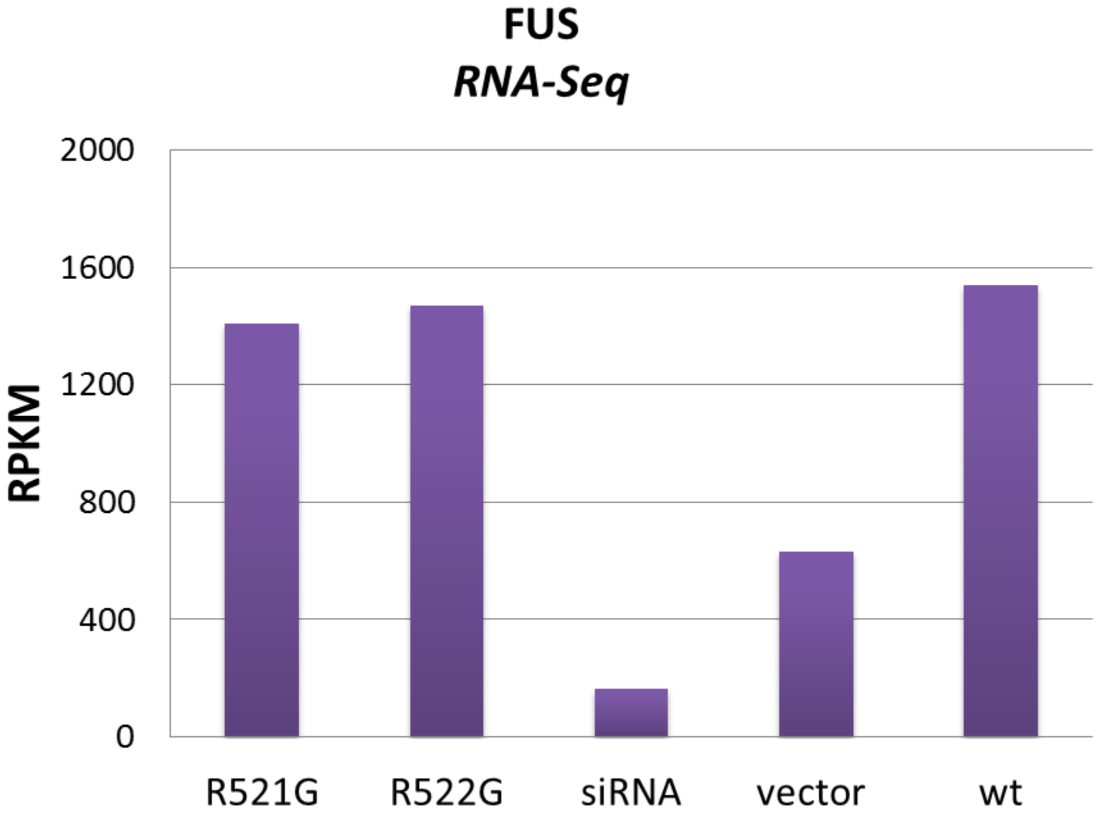

Figure S3. FUS protein levels measured by western blot.

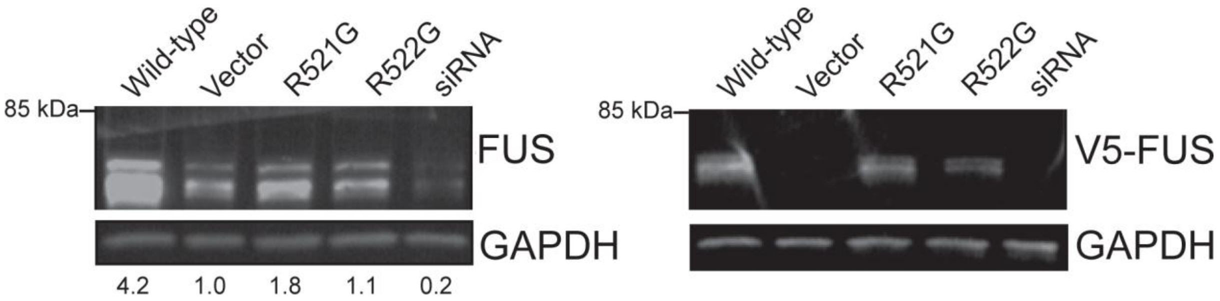

Figure S4. Correlation of RNA-Seq and RT-PCR for *RBM25*, *TAF15*, *TARS*, *TPR*, *APP* and *HN1*. Shown are the p-values for RNA-Seq and RT-PCR.

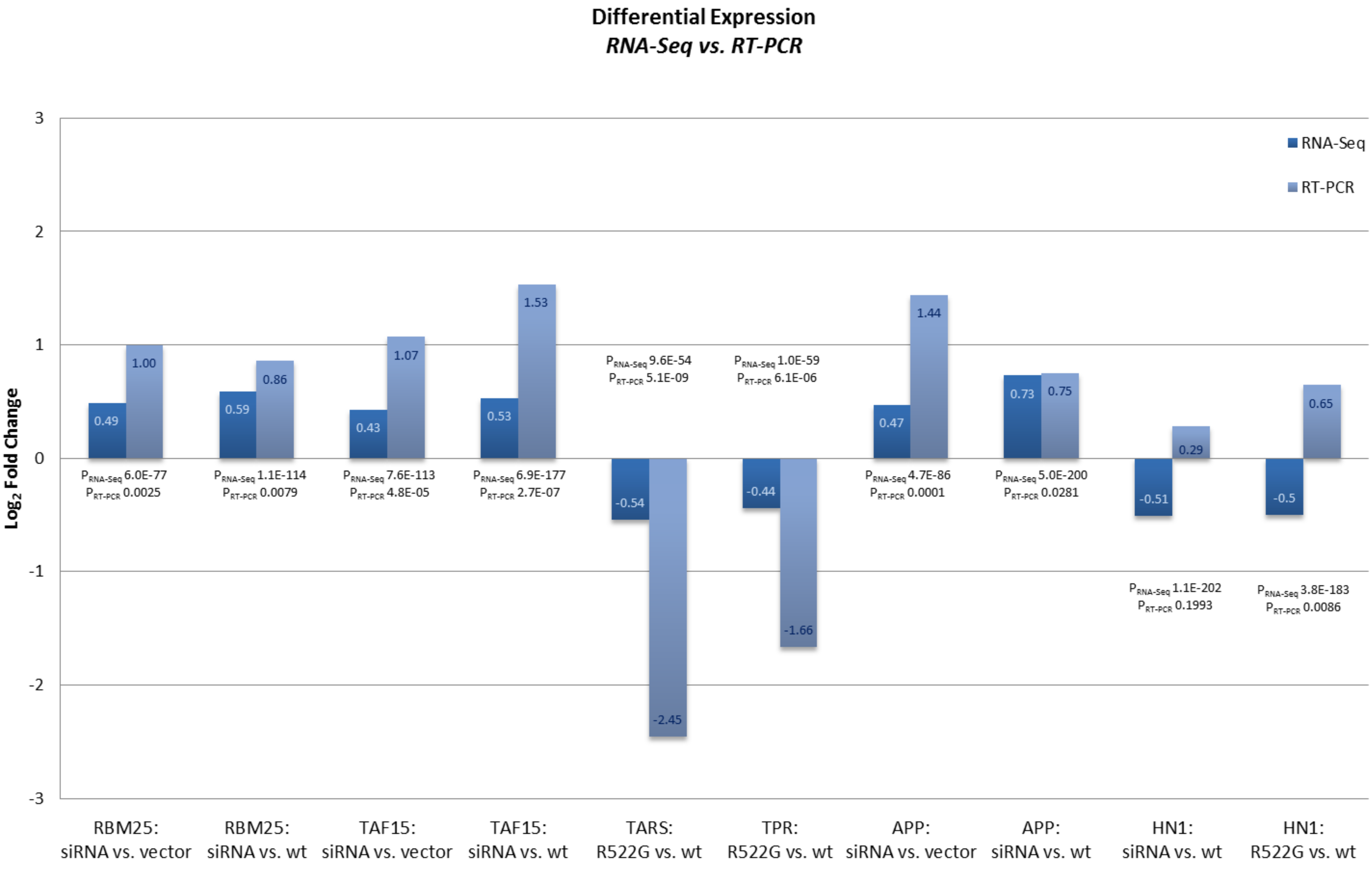

Figure S5. Correlation RNA-Seq and Semi-quantitative PCR for *PRPF8* and *RPS24*. Shown are the p-values for RNA-Seq and RT-PCR.

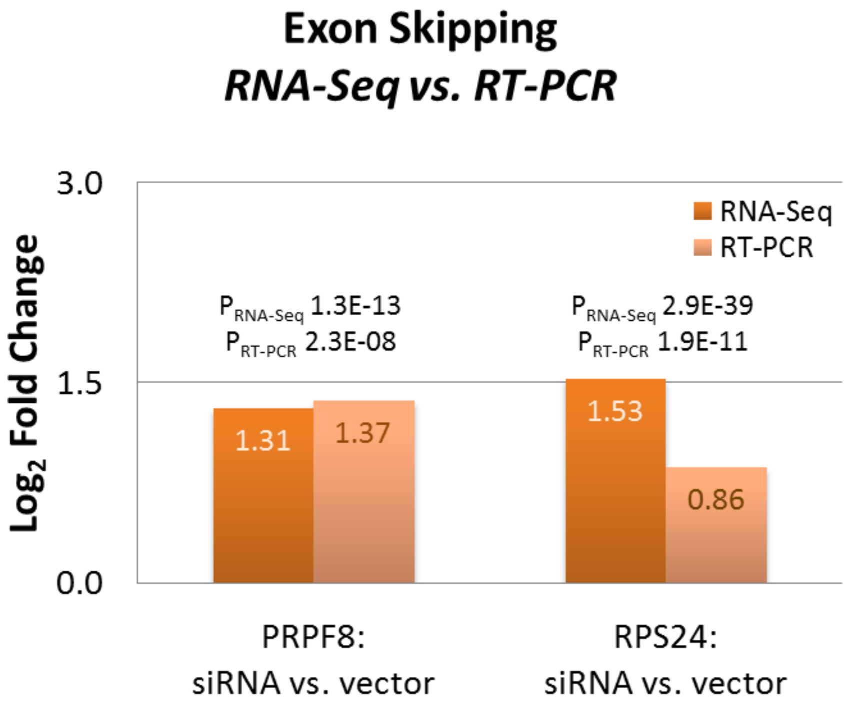

Supplement: File S1 — This file includes supporting materials and methods, Tables S1–S5, and Figures S1–S5. Legends for Figure S1–S5 are as follows: Figure S1. MA plots per condition comparison. MA plots per comparison where M = log (rpkmcondition) – log (rpkmvector) and A = 0.5 * (log (rpkmcondition)+log (rpkmvector)). Figure S2. FUS gene expression levels measured by RNA-Seq. Reads per kilobase of exon model per million uniquely mapped reads (RPKM) counts are displayed for each condition. Transfections with wild-type FUS, R521G and R522G resulted in a ∼2-fold increase in expression level, transfections with siRNA in a ∼4-fold decrease in expression level. Figure S3. FUS protein levels measured by western blot. The same samples were used for western blotting and RNA-Seq. We stained for FUS and the V5-tag (Materials and Methods), and bands were subsequently detected and quantified with an Odyssey Infrared Imaging System. For FUS, we compared each condition to the vector and calculated relative integrated intensities. We demonstrated that siRNA caused a decrease in FUS expression (1.0 versus 0.2), whereas transfections with wild-type FUS and the two mutants caused an increase in FUS expression (1.0 versus 4.2, 1.8 and 1.1). The multiple bands detected by FUS antibodies have been observed previously [51]. Figure S4. Correlation of RNA-Seq and RT-PCR for RBM25 , TAF15 , TARS , TPR , APP and HN1 . We selected 10 differentially expressed conditions within six genes for RT-PCR validation. These genes required at least one comparison with a log2 fold change of 0.4 (1.32 fold change). For each comparison, the log2 fold change and corresponding P-values are shown. Validation was observed in 8 out of 10 comparisons for 5 out of 6 genes. Figure S5. Correlation of RNA-Seq and Semi-quantitative PCR for PRPF8 and RPS24 . Two alternatively spliced genes, pre-mRNA-processing-splicing factor 8 (PRPF8) and ribosomal protein S24 (RPS24), with a log2 fold change in exon exclusion:inclusion above 0.4, (1.32 [file pone.0060788.s001.pdf]
